# Hidden phonon highways promote photoinduced interlayer energy transfer in twisted transition metal dichalcogenide heterostructures

**DOI:** 10.1126/sciadv.adj8819

**Published:** 2024-01-24

**Authors:** Amalya C. Johnson, Johnathan D. Georgaras, Xiaozhe Shen, Helen Yao, Ashley P. Saunders, Helen J. Zeng, Hyungjin Kim, Aditya Sood, Tony F. Heinz, Aaron M. Lindenberg, Duan Luo, Felipe H. da Jornada, Fang Liu

**Affiliations:** ^1^ Department of Materials Science and Engineering, Stanford University, Stanford, CA 94305, USA.; ^2^ SLAC National Accelerator Laboratory, Menlo Park, CA 94025, USA.; ^3^ Department of Chemistry, Stanford University, Stanford, CA 94305, USA.; ^4^ Stanford Institute for Materials and Energy Sciences, SLAC National Accelerator Laboratory, Menlo Park, CA 94025, USA.; ^5^ Department of Applied Physics, Stanford University, Stanford, CA 94305, USA.; ^6^ Stanford PULSE Institute, SLAC National Accelerator Laboratory, Menlo Park, CA 94025, USA.

## Abstract

Vertically stacked van der Waals (vdW) heterostructures exhibit unique electronic, optical, and thermal properties that can be manipulated by twist-angle engineering. However, the weak phononic coupling at a bilayer interface imposes a fundamental thermal bottleneck for future two-dimensional devices. Using ultrafast electron diffraction, we directly investigated photoinduced nonequilibrium phonon dynamics in MoS_2_/WS_2_ at 4° twist angle and WSe_2_/MoSe_2_ heterobilayers with twist angles of 7°, 16°, and 25°. We identified an interlayer heat transfer channel with a characteristic timescale of ~20 picoseconds, about one order of magnitude faster than molecular dynamics simulations assuming initial intralayer thermalization. Atomistic calculations involving phonon-phonon scattering suggest that this process originates from the nonthermal phonon population following the initial interlayer charge transfer and scattering. Our findings present an avenue for thermal management in vdW heterostructures by tailoring nonequilibrium phonon populations.

## Teaser

A unique photoinduced thermal transport channel at bilayer interfaces is shown to enhance thermal exchange.

## INTRODUCTION

Stacking atomically thin crystal layers into van der Waals (vdW) heterostructures (HSs) offers an exciting approach to create materials with unusual electronic, optical, and thermal properties beyond those of the constituent monolayers (*1*–*5*) and opens possibilities for promising applications in nanoelectronics, photonics, spintronics, and valleytronics (*6*–*12*). Heterobilayers based on transition metal dichalcogenides (TMDCs) are of particular interest, as they exhibit unique features of ultrafast interlayer charge transfer (CT) and a rich landscape of intra- and interlayer excitons (*3*, *4*, *13*–*18*), showing great potential for next-generation optoelectronic and excitonic applications (*4*, *11*, *12*, *19*, *20*). Moreover, the twist angle θ between the crystal lattice of adjacent layers in HSs provides an additional degree of freedom capable of further modulating their properties (*21*, *22*). The processes of charge carrier generation, transfer, recombination, and relaxation are strongly associated with phonon scattering and coupling (*23*–*28*). Phonons, as the main heat carriers in TMDCs, transport heat across the atomically sharp interface and hence determine the energy dissipation as well as thermal management in vdW HSs. A microscopic understanding of interfacial heat transfer and the management of nanoscale thermal transport is of utmost importance from both the fundamental and technological perspectives (*29*–*31*).

Many TMDC/TMDC HSs exhibit a type II band alignment (*32*, *33*), where their conduction band minimum and valence band maximum reside in opposite layers. Upon excitation of the HS by light, this alignment favors an interlayer CT, resulting in spatially separated but bound interlayer excitons. The interlayer CT has been reported to occur on a sub–100-fs timescale (*34*–*37*), independent of the interlayer twist angle (*35*, *37*). Several theoretical studies suggest phonon-mediated scattering plays a substantial role in the CT process via strongly hybridized intervalley excitons (*25*–*28*). However, despite considerable progress on the ultrafast CT in TMDC HSs, little work has been devoted to the photoinduced phonon dynamics in vdW HSs. This situation reflects the fact that conventional spectroscopic techniques, such as photoluminescence, transient absorption spectroscopy, and time- and angle-resolved photoemission spectroscopy, are not directly sensitive to phonon populations. By contrast, ultrafast electron diffraction (UED) is an ideal structural probe tool that allows direct measurement of real-time lattice dynamics with high temporal resolution and has been successfully applied to atomically thin two-dimensional (2D) materials (*38*–*42*). Recent UED studies on 15° WSe_2_/WS_2_ and MoS_2_/graphene HSs revealed that the ultrafast transfer and scattering of charges in the HS leads to sub-picosecond, nearly simultaneous, heating of both layers at early times (*40*, *41*, *43*). These results illustrate the complex physical phenomena that can arise from the light-induced lattice dynamics of a 2D HS. Here, we use mega–electronvolt (MeV) UED to probe systematically the light-induced lattice dynamics in macroscopic type II TMDC heterobilayers formed between MoX_2_ and WX_2_ (X: S and Se), at various twist angles. By selective excitation of MoX_2_ layer, we resolve real-time, layer-specific information of the heat exchange and thermalization process in the HSs.

## RESULTS

**Fig. 1.**
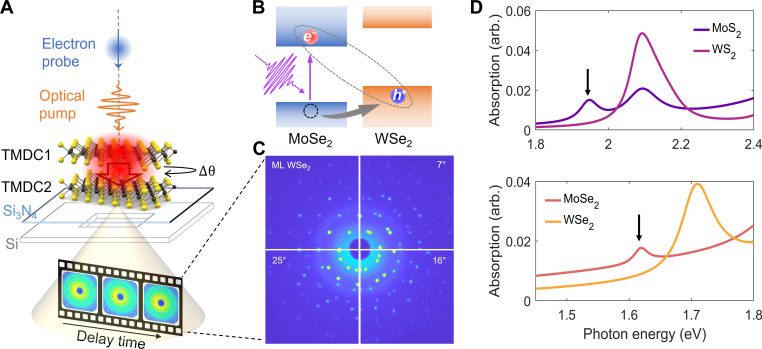
Overview of UED setup. (**A**) Schematic representation of the femtosecond optical pump, electron probe experiment, recording lattice dynamics of a TMDC heterostructure on a Si_3_N_4_ membrane. (**B**) Band alignment and carrier transfer for the type II heterobilayers excited on resonance with the MoS_2_ or MoSe_2_ A exciton. (**C**) Static diffraction image of the WSe_2_ monolayer (ML) and WSe_2_/MoSe_2_ heterobilayer with twist angles of 7°, 16°, and 25°. (Diffraction images for the other monolayers and heterobilayers are shown in fig. S2.) (**D**) Optical absorption of TMDC monolayers at 50 K. Top: MoS_2_ (purple) and WS_2_ (pink). Bottom: MoSe_2_ (red) and WSe_2_ (orange). The absorption spectra were obtained from fitting reflection contrast spectra. Arrows indicate the photon energy of the A exciton resonance of MoS_2_ (1.93 eV) and MoSe_2_ (1.62 eV).

The electron diffraction experiments were conducted using the MeV-UED facility at SLAC National Accelerator Laboratory (*44*, *45*). Figure 1A shows a schematic of the pump-probe UED experiment. In brief, an ultrafast pump laser with a pulse duration of ~60 fs resonantly excites the A excitons of MoX_2_. The type II band alignment allows holes to transfer from the MoX_2_ to the WX_2_ layer as shown in Fig. 1B, followed by phonon emission and transport between the layers. The layer-resolved lattice dynamics of the heterobilayer after photoexcitation are probed by diffraction using a ~150-fs, 4.2-MeV electron pulse. A few examples of static electron diffraction images of the monolayers and heterobilayers are shown in Fig. 1C. The large-area MoS_2_, WS_2_, MoSe_2_, and WSe_2_ monolayers were prepared using gold tape exfoliation (*46*), stacked at designed twist angles, and transferred to 10- to 15-nm-thick Si_3_N_4_ membrane window grids for Transmission Electron Microscopy (TEM). The twisted heterobilayers are uniform over the 250 μm–by–250 μm Si_3_N_4_ membrane (fig. S1). They allow clear identification of up to 10 orders of Bragg peaks in the diffraction images. The twist angles are determined by examination of the relative orientation θ between two sets of Bragg peaks, for which the actual twist angle between the two layers can be either θ or 60°-θ, due to symmetry. The optical absorption spectra for the monolayers (Fig. 1D) are used to identify the phonon energy required for resonant optical excitations. The samples are kept at cryogenic temperature (40 to 50 K) during the UED measurements.

**Fig. 2.**
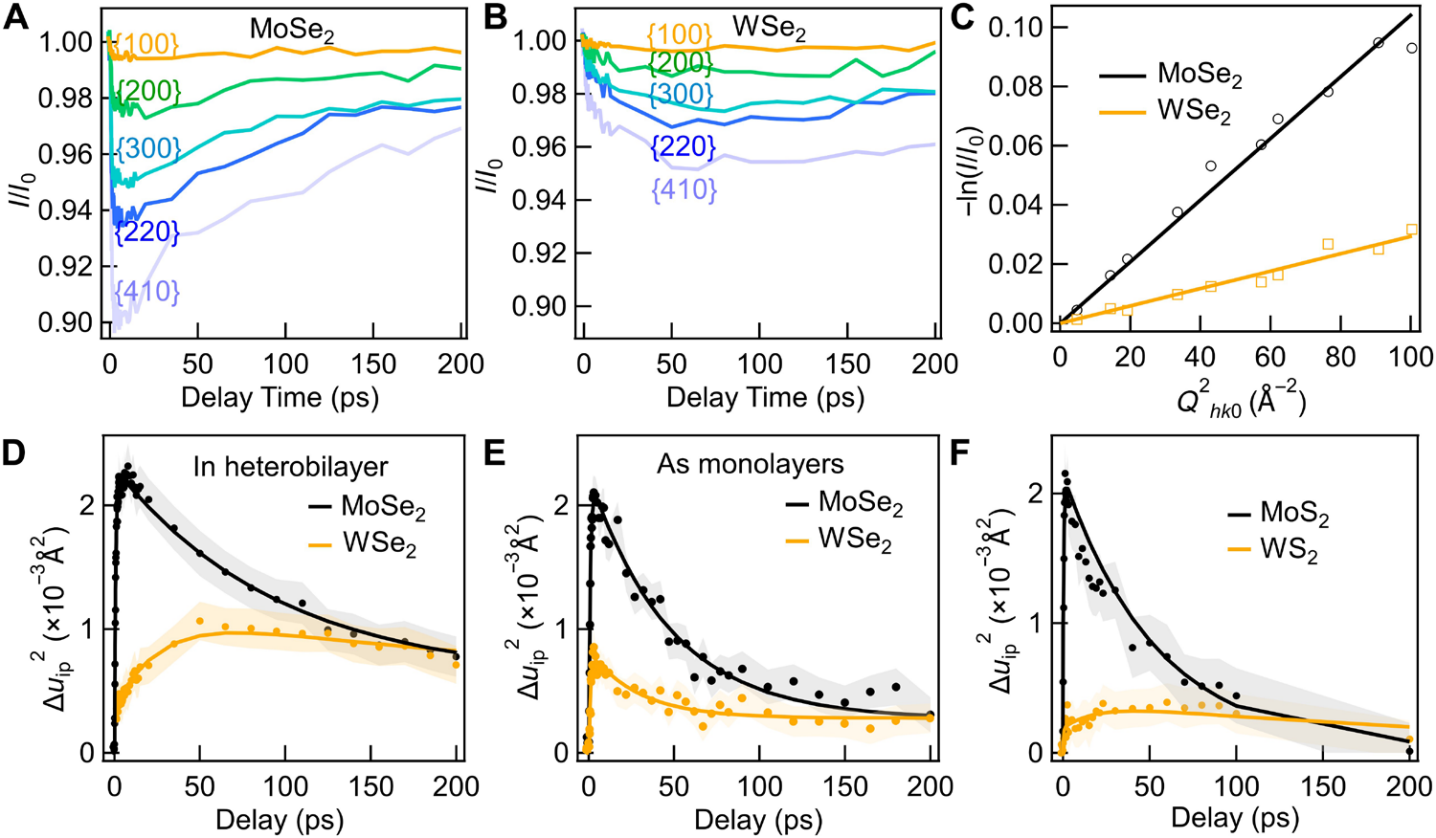
Layer-resolved Debye-Waller response. (**A** and **B**) Integrated Bragg peak intensities as a function of pump-probe delay time for different orders of Bragg peaks in the MoSe_2_ and WSe_2_ layers of a 25° WSe_2_/MoSe_2_ heterobilayer. The heterobilayer pumped on resonance with the MoSe_2_ A exciton with a fluence of 1 mJ/cm^2^. Ten different diffraction orders were used in the analysis. To avoid congestion, five representative diffraction orders are shown. Bragg order indexes and corresponding $Q_{\mathrm{hk}0}^{2}$ are listed in table S1. (**C**) Log intensity change for different orders of Bragg peaks at delay time of 12 ps, plotted against the reciprocal lattice vector of the Bragg peak squared, with the corresponding linear fit of the Debye-Waller model. (**D**) Mean square displacement (MSD) of the WSe_2_ and MoSe_2_ in the heterobilayer as a function of delay time. The solid lines are fit curves, and shaded area represents uncertainty. (**E**) MSD of isolated WSe_2_ and MoSe_2_ monolayers under the same optical pumping conditions. (**F**) MSD of layers in a 4° MoS_2_/WS_2_ heterobilayer as a function of delay time, pumped on resonance with the MoS_2_ A exciton under similar experimental conditions.

When photoexcitation creates electrons and holes in TMDC vdW HSs, phonons will be emitted through electron-phonon coupling during charge carrier scattering and relaxation. After that, further heat exchange and thermalization will occur between the layers. Experimentally, the Bragg peak intensities decrease upon photoexcitation because of greater thermal motion of the atoms in the lattice (*47*). The Bragg peak intensities of different orders in WSe_2_/MoSe_2_ heterobilayers at different delay times *t* after photoexcitation are shown in Fig. 2 (A and B). Similar results for MoS_2_/WS_2_ is presented in fig. S3. The reduction in the Bragg peak intensity can be modeled with the standard Debye-Waller theory in a 2D lattice as

$$-\text{ln}\left[\frac{I_{hk0}\left(t\right)}{I_{hk0}^{0}}\right]=\frac{1}{2}∆\langle u_{\text{ip}}^{2}\left(t\right)\rangle \cdot Q_{\mathrm{hk}0}^{2}$$ (1)

Here, $I_{\mathrm{hkl}}^{0}$ and *I*_*hk*0_(*t*) are the integrated Bragg peak intensities at pump-probe delay times of *t* < 0 and *t*, respectively. *Q*_*hk*0_ is the reciprocal lattice vector of the relevant Bragg peak, and $∆\left\langle u_{\text{ip}}^{2}\right\rangle$ is the increase of the in-plane mean square displacement (MSD) of atoms from their equilibrium positions due to the induced disorder (*38*, *41*). A good linear fit of $-\text{ln}\left[\frac{I_{hk0}\left(t\right)}{I_{hk0}^{0}}\right]$ versus $Q_{\mathrm{hk}0}^{2}$, as shown in Fig. 2C (WSe_2_/MoSe_2_) and fig. S3 (MoS_2_/WS_2_), reflects the validity of this approach for our measurement conditions. Twice the inferred slope then yields the value of change in MSD, $∆\left\langle u_{\text{ip}}^{2}\right\rangle$. The evolution of the MSD as a function of time is obtained by fitting the data at each delay time *t*.

One example for atomic displacements in the WSe_2_/MoSe_2_ HS is shown in Fig. 2D. Upon resonant excitation of the MoSe_2_ layer, the MSD of both layers rises immediately. At later times, the MSD falls due to heat dissipation into the underlying Si_3_N_4_ substrate and surrounding Si substrate, which act as heat sinks (*48*). The overall time-dependent MSD can be fitted correspondingly with a fast single-exponential rise and a multiexponential decay (convolved with the instrument response function). The detailed fitting procedure is described in the Supplementary Materials. Because the MoSe_2_ layer is under direct photoexcitation, its lattice heats up very rapidly through efficient electron-phonon interactions. The time constant τ_1_ for this prompt rise is 1 ps or shorter, which is also in good agreement with previously reported UED measurements of TMDC monolayers and bilayers (*38*, *40*, *41*, *43*, *49*). We observe a similar rapid rise for photoexcitation of isolated MoSe_2_ and WSe_2_ monolayers (Fig. 2E).

In contrast to the sub-picosecond lattice heating in the MoSe_2_ layer, the MSD in the WSe_2_ layer of the heterobilayer exhibits an additional, slower component of growth. The heat flowing into the WSe_2_ layer has contributions from: (i) ultrafast interlayer hole transfer from MoSe_2_ to WSe_2_, creating phonons through electron-phonon coupling: This is expected to occur on the sub-picosecond timescale, as verified by earlier experimental results and first-principles calculations (*40*, *41*, *43*); (ii) weak optical absorption in the tail of the WSe_2_ A exciton peak: This contribution should also lead to sub-picosecond response, as seen in isolated WSe_2_ monolayer in Fig. 2E; and (iii) interlayer heat transfer from MoSe_2_ through anharmonic phonon-phonon interactions, which may occur at longer times.

In keeping with this discussion, the time-dependent MSD of WSe_2_ plotted in Fig. 2D was fit with two different exponential rise components with fast (τ_1_) and slow (τ_2_) time constants. The fast rise τ_1_ for WSe_2_ layer is ~0.5 to 1 ps, similar to that of the MoSe_2_ layer and of the isolated WSe_2_ monolayers. This time constant is consistent with previous reports of the WS_2_/WSe_2_ HS, which revealed almost simultaneous heating of both layers in the HS, facilitated by the ultrafast interlayer CT (*40*, *41*, *43*). In contrast, the longer time constant τ_2_, which reflects the process of the WSe_2_ layer gaining additional energy from the adjacent photoexcited MoSe_2_ monolayer, is on the order of 10 to 30 ps. This slow thermal heating channel is unique to the WSe_2_ in a heterobilayer and is not observed in isolated monolayers. Similarly, we also identify a slow heating channel for the WS_2_ layer in MoS_2_/WS_2_ heterobilayers, arising from heat flow from the photoexcited MoS_2_ layer (shown in Fig. 2F).

**Fig. 3.**
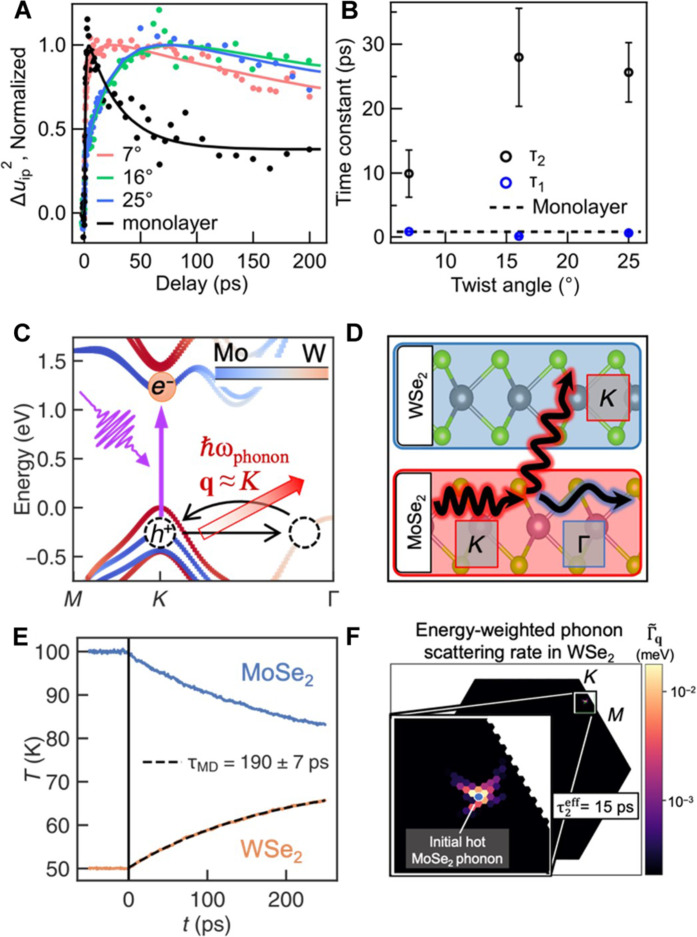
Interlayer phonon scattering. (**A**) MSD of the WSe_2_ layer in WSe_2_/MoSe_2_ HSs with twist angles of 7° (red), 16° (green), and 25° (blue) and of the WSe_2_ monolayer (black). The MSD curves are normalized to their maximum fit values to aid in visualizing the different rise dynamics across samples. (**B**) Inferred values for the fast (τ_1_, blue) and slow (τ_2_, black) rise time constants for WSe_2_ in the HS as a function of twist angles. The black dashed line represents τ_1_ of the monolayer. (**C**) Layer-projected electronic band structure of a MoSe_2_/WSe_2_ HS showing a type II band alignment. After resonantly exciting the MoSe_2_ A exciton, the exciton-bound holes at the *K* and *K*′ valleys scatter to layer-hybridized states around the Γ valley by emitting phonons with wave vectors close to *K* or *K*′. Eventually, the holes scatter back to the *K* and *K*′ valley states localized in WSe_2_. (**D**) Diagrammatic representation of the fission-like interlayer phonon scattering. A higher-energy *K* wave vector phonon in the MoSe_2_ layer decays into two lower-energy phonons: a lower energy near-Γ wave vector acoustic phonon (blue) in the MoSe_2_ layer and a higher energy near-*K* wave vector acoustic phonon in the adjacent WSe_2_ layer (red). (**E**) Interlayer thermalization time from MD simulations assuming each layer is individually in a local thermal equilibrium. (**F**) Brillouin zone (BZ)–resolved thermal scattering rate computed for the fission diagram in (D). Weighted scattering rate ${\tilde{\Gamma }}_{q}$ are calculated as the fraction of energy transferred from the initial MoSe_2_ phonon with wave vector **q** lying between *K* and *M* (the blue dot at the center of the inset) to various phonons on WSe_2_ (the **q** → 0 acoustic phonon left behind on MoSe_2_ is not shown).

The dependence of interfacial thermal transfer on different twist angles is important for elucidating the light-induced thermal dynamics in TMDC heterobilayers, especially those within moiré structures. Figure 3A shows the normalized MSD of the WSe_2_ layer in WSe_2_/MoSe_2_ HSs at twist angles of 7°, 16°, and 25° and in WSe_2_ monolayers. The corresponding values of τ_1_ and τ_2_ are shown in Fig. 3B. Among the tested samples with different twist angles, the time constant τ_2_ of the WSe_2_ layer appears slightly longer in the 16° and 25° heterobilayers compared with that in the 7° heterobilayer. This suggests a slower interlayer thermal energy transfer rate as the twist-angle mismatch increases. On the other hand, the fast response τ_1_ remains 1 ps or less in the MoSe_2_ and WSe_2_ layers for all the HSs with different twist angles.

A layer-projected density functional theory (DFT) calculation of the electronic band structure of a MoSe_2_/WSe_2_ heterobilayer is displayed in Fig. 3C, showing the expected type II band alignment (see the Supplementary Materials). After resonantly exciting the MoSe_2_ A exciton, the exciton-bound holes at the *K* and *K*′ valleys scatter to layer-hybridized states around the Γ valley. Eventually, the holes scatter back to the *K* and *K*′ valley states localized in WSe_2_, forming interlayer excitons (*8*, *43*, *50*). Overall, this interlayer CT occurs on the order of a few tens of femtoseconds (*24*, *26*, *51*), independent of twist angles (*8*, *37*). The scattering and energy relaxation of holes will create an initial distribution of phonons in both the MoSe_2_ and WSe_2_ layers, resulting in a sub-picosecond rise of the MSD. Experimentally, we measured a sub-picosecond τ_1_ in both heterobilayers and monolayers.

Throughout the ultrafast charge creation, relaxation and transfer between the two layers, any asymmetry in the phonon emission will lead to a temperature asymmetry between the layers. Such an interfacial temperature asymmetry would lead to a subsequent heat transfer between the layers, which explains the slower heating (τ_2_ time constant) of WSe_2_ seen in our experiments. To gain a microscopic understanding of the heat transfer process and obtain a quantitative assessment of the temperature in our experiments, we perform a series of atomistic calculations. We first performed molecular dynamics (MD) simulations using first-principles–parameterized empirical force fields with the Large-scale Atomic/Molecular Massively Parallel Simulator (LAMMPS) code (see the Supplementary Materials) (*52*). These simulations on MoSe_2_/WSe_2_ heterobilayers reveal the expected linear relationship between each layer’s temperature and its MSD (fig. S4) and predict peak temperatures in our experiments close to 100 K immediately after the MoSe_2_ layer is optically pumped. While such classical calculations overestimate the interfacial thermal boundary conductance by using classical phonon statistics, the timescale associated with the heat conductance is often qualitatively well-described within a classical picture (*53*).

We first assume that each layer in the heterobilayer system is individually in a local thermal equilibrium and subsequently let the system reach its global thermal equilibrium, as shown in Fig. 3E. On the basis of these assumptions, we deduce an interlayer thermalization time τ_MD_ between 190 and 350 ps. The timescale is consistent with thermalization time estimated from the previously reported TMDC interfacial thermal conductivities (*43*). However, it is about one order of magnitude longer than that τ_2_ measured from experiments. The computed thermalization times also display a weak dependence on the twist angles we considered, from 7° to 25° (fig. S5). For the smaller twist angles where moiré structures are prominent, our simulations were not large enough to capture the large-scale relaxation domains and soliton formations.

To gain a deeper understanding of the limitations of the initial simulations, which assumed that the two layers were thermalized subsystems, it is important to consider the strict phase space constraints associated with the decay path of excited electrons and holes. As a result, the initially created phonons are restricted to crystal momenta close to a few high symmetry points in the Brillouin zone (BZ) (*51*, *54*). The momentum-dependent electron-phonon interactions and specific inelastic electron-phonon scattering pathways for hot carrier relaxation lead to a profoundly nonequilibrium phonon state with anisotropic momentum distribution. In particular, when the excited holes of MoSe_2_ at the *K* and *K*′ valleys scatter to the regions around the Γ valley, primarily acoustic phonons are emitted whose wave vectors **q** ≈ *K* form a ring around *K* in momentum space. This can be understood in terms of energy-momentum conservation arguments (*43*). Such phonons are localized in the MoSe_2_ layer where the initial hole resides (see the Supplementary Materials). This mechanism is universal for different stackings of TMDC heterobilayers (*8*, *24*), leading to a nonthermal phonon distribution.

This nonequilibrium state may persist for a certain duration after photoexcitation. Prior UED and diffuse scattering experiments have shown that the phonon distribution of MoS_2_ monolayers remains anisotropic within the initial 5 ps following photoexcitation (*39*). In addition, first-principles calculations of the electron-phonon and phonon-phonon interactions in monolayer MoS_2_ revealed that the nonequilibrium anisotropic distribution of phonons can endure for 10 ps or longer (*51*). Further thermalization and relaxation of the anisotropic nonequilibrium phonons involve couplings within the monolayer, between the monolayers, and between the HS and the substrate.

To investigate the role of such nonequilibrium phonons in the apparent heating of the adjacent layer (WSe_2_), we evaluate the mode-resolved, three-phonon scattering matrix elements by computing the anharmonic, third-order force constants using a supercell approach (*55*) in a commensurate HS with 0° twist angle. We restrict our analysis here to the study of the phonon scattering from MoSe_2_ to WSe_2_ in bilayer MoSe_2_/WSe_2._ This enabled us to determine the characteristic timescales associated with the thermalization of an initially excited phonon in MoSe_2_. The interfacial phonon-mediated heat transfer will be primarily driven by interlayer phonon-phonon scattering. Therefore, we extract the interfacial scattering processes by calculating the phonon-phonon interlayer matrix elements in a layer-resolved basis using a separable subsystem approach (*56*). Specifically, we compute the coupling between a MoSe_2_ phonon to any pairs of phonons on MoSe_2_ and/or WSe_2_ by rotating the force constants explicitly computed on the bilayer system onto a product of monolayer phonon basis (see the Supplementary Materials). Given an initial nonthermal distribution of phonons within the expected wave vector range around *K*, our calculations demonstrate that the fastest interfacial phonon-phonon scattering events occur within a time range of 15 to 40 ps.

Figure 3D shows a diagram for a representative fast interfacial phonon scattering pathway, in which a **q** ≈ *K* phonon in MoSe_2_ undergoes a fission-like decay into two phonons: a low-energy **q** ≈ Γ phonon in the MoSe_2_ layer and a high-energy **q** ≈ *K* phonon in the WSe_2_ layer, transferring energy to the WSe_2_ layer. In this representative pathway, the initial **q** ≈ *K* phonons within the MoSe_2_ layer lie on the acoustic branch and show primarily an in-plane chiral motion of the chalcogen atoms and, to a lesser extent, an out-of-plane displacement of the transition-metal atoms (see the Supplementary Materials). We find that such fission-like processes emit a long wavelength acoustic phonon, primarily within the original MoSe_2_ layer, and an additional higher-energy **q** ≈ *K* phonon in the WSe_2_ layer. The momentum-resolved scattering-rate associated with such final WSe_2_ phonons is shown in Fig. 3F. These larger–wave vector WSe_2_ phonons carry most of the energy during the fission of initial MoSe_2_ phonons, leading to the interlayer thermalization. By integrating the scattering rate over the final distribution of phonons emitted on the WSe_2_ layer, we find the fastest (and rate-dominating) channels yield a minimum effective scattering time of $\tau_{2}^{\text{eff}}\approx \;15$ ps, which is in good agreement with our experimental results. Our microscopic analysis also reveals that fast interlayer phonon scattering occurs due to the presence of nearly degenerate phonon modes around **q** ≈ *K* on both layers, which can interact via the absorption or emission of low-energy, near-zero momentum, and layer-hybridized acoustic phonons. This three-phonon scattering process is possible in bilayer MoSe_2_/WSe_2_ because the acoustic phonons at the BZ boundary primarily involve the motion of the chalcogen atoms within each monolayer. Hence, the relevant acoustic phonons near **q** ≈ *K* on MoSe_2_ and WSe_2_ overlap in momentum and energy (see the Supplementary Materials and fig. S6). In contrast, HSs such as WS_2_/WSe_2_ studied previously with UED (*40*, *41*, *43*) do not display nearly degenerate phonons near **q** ≈ *K* but rather a 30 cm^−1^ energy gap in the relevant ZA modes. The lack of scattering channels may be one reason why the slow rise time constant τ_2_ was not previously observed (see the Supplementary Materials).

Compared with interfacial phonon scattering from MoSe_2_ to WSe_2_, we also find the intralayer phonon scattering within the MoSe_2_ layer is approximately five times faster. However, such intralayer phonon scattering channels involve the emission of **q** → 0 phonons with a characteristic root mean square distribution in reciprocal space that is only 3% that of phonons at the BZ edge. Hence, we expect the initial nonthermal distribution of **q** ≈ *K* phonons in MoSe_2_ to be relatively localized in momentum space for a period long enough for the interlayer phonon scattering to take place. We also note that our calculations of nonequilibrium interfacial heat transfer are performed with 0° twist angle. For small but finite twist angles, the formation of large-scale moiré stacking domains likely functions as a stacking fault and serves as phonon scattering centers mimicking the result of the commensurate structure.

## DISCUSSION

In conclusion, we have used UED to trace directly the layer-resolved lattice heating dynamics as charge and energy are transferred across vdW interfaces in MoS_2_/WS_2_ and WSe_2_/MoSe_2_ HSs. Following the fast electron-phonon interactions that heat the lattice on a sub-picosecond timescale, interfacial heat transfer occurs on the timescale of tens of picoseconds due to phonon-phonon interactions. The corresponding rate of heat exchange is an order of magnitude faster than that predicted by MD simulations for a fully thermalized model. This difference is attributed to the nonthermal, highly polarized distribution of phonons generated by the initial charge carrier scattering and interlayer CT. Such a nonthermal distribution of phonons leads to a time constant for heat flow within a perturbative theoretical analysis of about 15 ps, comparable to experiment and substantially faster than that for a phonon distribution that is thermalized within a single layer. The interfacial lattice dynamics and the possibility of fast phonon-phonon scattering channels revealed in this work are essential components in the mechanisms of energy transfer in TMDC heterobilayers. This unique mechanism of heat exchange under photoexcited nonequilibrium conditions is of particular interest in the thermal management of the optoelectronic and photonic devices using TMDC HSs in the future.

## MATERIALS AND METHODS

### Experimental UED setup

Ultrafast pump-probe experiments on the TMDC/TMDC HSs were conducted in the MeV-UED facility at SLAC National Accelerator Laboratory. The femtosecond electron pulses are at normal incidence in a transmission mode geometry. The details on the MeV-UED apparatus can be found elsewhere (*44*, *45*). Electron pulses with 4.2-MeV kinetic energy generated from a photocathode radio-frequency gun were used for the experiment. The MeV electron pulse at the sample position has <150-fs full width at half maximum (FWHM) pulse duration and ~200-μm FWHM spot size. Pump laser pulses near collinearly illuminated the sample together with probe electron pulses. A Ti:sapphire regenerative and multipass amplifier laser system delivers 800-nm laser pulses with 60-fs FWHM pulse duration. Most of the 800-nm laser is used to drive an optical parametric amplifier to generate the resonant pump beam for different TMDC/TMDC HSs. The HSs are cooled down to 40 to 50 K for the cryogenic measurements and 300 K for the room temperature measurements. The diffracted patterns were recorded with a phosphor screen and a lens-coupled ANDOR iXon Ultra 888 electron-multiplying charge-coupled device camera. The pump-probe measurement was performed at a 360-Hz repetition rate. Diffraction images were recorded and averaged at various pump-probe time delays, which provides a high signal-to-noise ratio to perform quantitative dynamics analysis.

### Sample preparation

Monolayer samples were prepared using the method of gold tape exfoliation as detailed in (*46*). A 100-nm-thick gold layer is deposited on a flat silicon substrate (NOVA Electronic Materials LLC) via electron-beam evaporation (Kurt J. Lesker LAB 18 e-beam evaporator). A layer of polyvinylpyrrolidone (PVP) solution (Sigma-Aldrich, *M*_w_ of 40,000 10 wt % in ethanol/acetonitrile wt 1/1) is spin-coated on top of the Au film (400 rpm, acceleration of 400 rpm/s, 2 min) and cured at 150°C for 5 min as a sacrificial layer to prevent tape residue contamination. The prepared PVP/Au is picked up with thermal release tape (TRT) (Semiconductor Corp.; release temperature, 90°C), revealing an ultraflat, clean, and fresh gold surface, i.e., the gold tape. The gold tape is pressed onto freshly cleaved bulk MoS_2_, WS_2_, MoSe_2_, or WSe_2_ single crystals (HQ Graphene). As the tape is lifted off the surface, it carries the PVP/Au layer with a monolayer attached to the Au surface. This is transferred to the 285-nm SiO_2_/Si substrate (NOVA Electronic Materials LLC), and the TRT is removed by heating at 95°C. The PVP layer is removed by dissolving it in deionized water via 15 min of continuous stirring. The Au layer is dissolved in a KI/I_2_ gold etchant solution [2.5 g of I_2_ and 10 g of KI in 100 ml of deionized (DI) water; iodine, 99.99% (Alfa Aesar); and potassium iodide, 99.9% (Alfa Aesar)]. The monolayer is rinsed with DI water and isopropanol and dried with N_2_. For stacking heterobilayers, the first layer is prepared on SiO_2_/Si. The second layer is exfoliated with a fresh piece of gold tape to obtain the TRT/PVP/Au/monolayer stack and is placed on the first layer at a desired twist-angle. The tape is released, and the heterobilayer then goes through the same rinsing and etching procedure as the first monolayer. The heterobilayers on SiO_2_/Si were soaked in acetone for 20 min to 4 hours to promote separation of the TMDC from the substrate before transfer. For transferring onto Si_3_N_4_ films, a cellulose acetate butyrate (CAB) (Sigma-Aldrich) solution in ethyl acetate was spin-coated on top of the heterobilayer (400 rpm, acceleration of 400 rpm/s, 2 min). The CAB-coated heterobilayer was then dipped in water at a shallow angle, and intercalation of water between the hydrophobic CAB/heterobilayer film and hydrophilic SiO_2_/Si substrate released the film from the substrate. The CAB/heterobilayer was then placed on 10- to 15-nm-thick 250 μm–by–250 μm Si_3_N_4_ membrane windows on Si TEM grids (Ted Pella; TEMWindows). The CAB was dissolved in ethyl acetate, leaving clean monolayer and heterobilayers on the Si_3_N_4_ film. Two example sample images are shown in fig. S1.

### Reflection contrast spectroscopy

To measure the optical absorption of the monolayers, the reflection contrast data were taken with a stabilized Tungsten-Halogen light source (Thorlabs SLS201L, 450 to 5500 nm) focused to ~1 μm on the sample by a 40× objective. The reflected light was then collected by the same objective and measured and analyzed by a Jobin Yvon iHR550 spectrometer equipped with a Synapse CCD camera. All reflection contrast measurements were conducted at ~40 K in a Montana Instrument cryostat. The reflection contrast data were acquired by calculating (*R*_sample_ − *R*_ref_)/*R*_ref_, where *R*_sample_ is the reflected light spectrum from the sample area and *R*_ref_ is the reflected spectrum from the TEM grid substrates. The absorption spectra were obtained by fitting the reflection contrast spectra using a transfer matrix method and the RefFit software (*57*).

### MoSe_2_/WSe_2_ electronic band structure calculation

The DFT electronic band structure for MoSe_2_/WSe_2_ in the present text is computed with Quantum ESPRESSO, a first-principles DFT, plane-wave–based pseudopotential code (*58*). We consider a MoSe_2_/WSe_2_ HS in the 0° twist angle, $R_{h}^{x}$ stacking and use a 6 × 6 × 1 *k*-point grid with an 80-rydberg energy cutoff and 20 Å of vacuum. We use a Perdew-Burke-Ernzerhof (PBE) exchange-correlation functional (*59*) with a vdW correction “vdw-df-c09x” within the framework of Dion *et al*. (*60*) and Cooper (*61*). We include spin-orbit coupling within a fully relativistic framework in this calculation. The square of this projection is used to color the bands in Fig. 3C.

### Thermalized bilayer MD simulations

To compute the heat transport between MoSe_2_ and WSe_2_ assuming a thermalized phonon distribution, we perform classical MD simulations. We construct several MoSe_2_/WSe_2_ lattice configurations with angles between 1° and 27°. For each twist angle, we choose an initial difference in temperatures $\Delta T=T_{0}^{\text{Mo}}-T_{0}^{W}=50\;K$, where $T_{0}^{\text{Mo}}=100\;K\;\text{and}\;T_{0}^{W}=50\;K$; these values are chosen to approximate those in the experimental setup when the MoSe_2_ layer is initially pumped. After a thermalization step of the individual layers, at *t* = 0, we release the two thermostats applied to the individual layers and let the bilayer thermalize as a whole (see Fig. 3E). We fit single exponentials to the rising WSe_2_ temperature, *T*^W^, versus time and plot the temperature rise constants τ^W^ at different twist angles in fig. S5. Further details are discussed in section S2.

### First-principles phonon calculation using a perturbation theory approach

To quantify the nonthermal interfacial heat transfer, we use a layer-separable basis approach in which we rotate the phonon-phonon scattering matrix into a basis in which we can directly characterize all phonon modes by layer. We used a similar approach to Ouyang *et al.* (*56*), which calculated the phonon-phonon scattering elements by the off-diagonal components of the bilayer dynamical matrix rotated into a monolayer basis, but we extended this approach here to include the anharmonic effects from three-phonon scattering processes. Further details are discussed in section S3.

## Supplementary Materials

### This PDF file includes:

Sections S1 to S4 Figs. S1 to S13 Table S1 References
