# Supplementary material for: Hidden phonon highways promote photoinduced interlayer energy transfer in twisted transition metal dichalcogenide heterostructures
Source: Sci Adv. 2024 Jan 24;10(4):eadj8819. doi: 10.1126/sciadv.adj8819 (PMC10807799; doi:10.1126/sciadv.adj8819)
Supplement: Supplementary file 1 — Sections S1 to S4 Figs. S1 to S13 Table S1 References [file sciadv.adj8819_sm.pdf]

Science Advances
AAAS

Supplementary Materials for

Hidden phonon highways promote photoinduced interlayer energy transfer in twisted transition metal dichalcogenide heterostructures

Amalya C. Johnson *et al.*

Corresponding author: Fang Liu, fliu10@stanford.edu; Duan Luo, luoduan@opt.ac.cn

Sci. Adv. **10**, eadj8819 (2024)
DOI: 10.1126/sciadv.adj8819

This PDF file includes:

Sections S1 to S4
Figs. S1 to S13
Table S1
References

Contents

1. Fitting models for time dependent MSD
2. Thermalized bilayer Molecular Dynamics simulations
3. First-principles phonon calculation using a perturbation theory approach
4. Supplementary figures and tables

1. Fitting Models for time dependent MSD

Two models can be used to represent the time-dependent response of the MSD. The first is a single exponential rise and double exponential decay model convolved with the instrument response function,

$$\begin{aligned}\langle u_{ip}^2\rangle = A\Bigg(&-\exp\left(-\left(\frac{t-t_0}{\tau_1}+\frac{\sigma}{2\tau_1^2}\right)\right)\left(\frac{1}{2}+\frac{1}{2}erf\left(\frac{t-t_0}{\sqrt{2}\,\sigma}-\frac{\sigma}{\sqrt{2}\tau_1}\right)\right)\\ &+(1-b)\exp\left(-\left(\frac{t-t_0}{\tau_3}+\frac{\sigma^2}{2\tau_3^2}\right)\right)\left(\frac{1}{2}+\frac{1}{2}erf\left(\frac{t-t_0}{\sqrt{2}\,\sigma}-\frac{\sigma}{\sqrt{2}\tau_3}\right)\right)\\ &+b\exp\left(-\left(\frac{t-t_0}{\tau_4}+\frac{\sigma^2}{2\tau_4^2}\right)\right)\left(\frac{1}{2}+\frac{1}{2}erf\left(\frac{t-t_0}{\sqrt{2}\,\sigma}-\frac{\sigma}{\sqrt{2}\tau_4}\right)\right)\Bigg)\end{aligned} \tag{1}$$

where A is the amplitude of the response, t_0 is the time of excitation, σ is the instrumental resolution, τ_1 is the time constant for the fast exponential rise, and τ_3 is the time constant for the fast exponential decay due to vertical heat dissipation. τ_4 is the time constant for the slow exponential decay due to lateral heat dissipation, and b is the contribution of τ_4 to the decay in comparison with τ_3. τ_4 is fixed to 6 ms as previously measured on WSe_2/$MoSe_2$ twisted bilayers on Si_3N_4 (*48*). Although our experimental time scale is not sensitive to such long time, it will add a non-zero offset to the end of the scan. This model well represents all monolayer MSDs and the MoS_2 and $MoSe_2$ layers of all heterobilayers.

For the WSe_2 and WS_2 layer of the heterobilayer, the model contained a fast exponential rise time constant (τ_1), a slow exponential rise time constant (τ_2), and the same fast exponential decay time constant (τ_3) and slow decay time constant (τ_4):

$$
\begin{aligned}
\langle u_{ip}^2 \rangle = A\Bigg(& -(1-a)\exp\left(-\left(\frac{t-t_0}{\tau_1}+\frac{\sigma}{2\tau_1^2}\right)\right)\left(\frac{1}{2}+\frac{1}{2}erf\left(\frac{t-t_0}{\sqrt{2}\,\sigma}-\frac{\sigma}{\sqrt{2}\tau_1}\right)\right) \\
& -a\exp\left(-\left(\frac{t-t_0}{\tau_2}+\frac{\sigma}{2\tau_2^2}\right)\right)\left(\frac{1}{2}+\frac{1}{2}erf\left(\frac{t-t_0}{\sqrt{2}\,\sigma}-\frac{\sigma}{\sqrt{2}\tau_2}\right)\right) \\
& +(1-b)\exp\left(-\left(\frac{t-t_0}{\tau_3}+\frac{\sigma^2}{2\tau_3^2}\right)\right)\left(\frac{1}{2}+\frac{1}{2}erf\left(\frac{t-t_0}{\sqrt{2}\,\sigma}-\frac{\sigma}{\sqrt{2}\tau_3}\right)\right) \\
& +b\exp\left(-\left(\frac{t-t_0}{\tau_4}+\frac{\sigma^2}{2\tau_4^2}\right)\right)\left(\frac{1}{2}+\frac{1}{2}erf\left(\frac{t-t_0}{\sqrt{2}\,\sigma}-\frac{\sigma}{\sqrt{2}\tau_4}\right)\right)\Bigg)
\end{aligned}
\tag{2}
$$

where a is the contribution of τ_2 to the rise over τ_1.

Since the time constants τ are longer than experimental resolution σ, the model can be simplified and gives similar best-fit parameters:

$$
\begin{aligned}
\langle u_{ip}^2 \rangle = A\Bigg(& -(1-a)\exp\left(-\left(\frac{t-t_0}{\tau_1}+\frac{\sigma}{2\tau_1^2}\right)\right)-a\exp\left(-\left(\frac{t-t_0}{\tau_2}+\frac{\sigma}{2\tau_2^2}\right)\right) \\
& +(1-b)\exp\left(-\left(\frac{t-t_0}{\tau_3}+\frac{\sigma^2}{2\tau_3^2}\right)\right)+b\exp\left(-\left(\frac{t-t_0}{\tau_4}+\frac{\sigma^2}{2\tau_4^2}\right)\right)\Bigg)
\end{aligned}
\tag{3}
$$

2. Thermalized Bilayer Molecular Dynamic Simulations

Mean square displacement and temperature

To quantitatively determine the Debye-Waller term, we perform molecular dynamics calculations to relate the temperature of individual $MoSe_2$ and WSe_2 monolayers to their in-plane mean-square displacements (MSD). To achieve this, we initialize both layers at the same temperature, $T_0^M = T_0^W$, from 40 K to 100 K, and subsequently calculate their final in-plane MSD at long timescales (1 ns). As shown in Fig. S4, the MSD displays the expected linear dependence on the temperature. Comparing Fig. S4 to the MSD peaks in Fig 2. near 0.01 Å^2 suggests peak in-plane temperatures near 100 K for both the $MoSe_2$ and WSe_2 monolayers after the $MoSe_2$ is optically pumped. We perform all MD simulations with the LAMMPS package incorporating both a Stillinger-Weber potential (*63*) for modeling interactions within a single TMD layer and Kolmogorov-Crespi (*64*) for capturing interlayer interactions, both of which are critical to capturing proper atomic relaxation in TMD bilayers.

3. First-Principles Phonon Calculation using a Perturbation Theory Approach

Phonon eigenvector and dispersion

We use the PHONOPY package to compute, within the harmonic approximation, the phonon frequencies $\omega_{\mathbf{q}\nu}$ and eigenvectors $\mathbf{e}_\nu(\mathbf{q})$ with crystal momentum wave vector $\mathbf{q}$ and branch index ν. These eigenvectors are obtained by diagonalizing the dynamical matrix constructed from the second-order force constants $\Phi_{\alpha\beta}(jl, j'l')$. The second-order force constants are defined as the change in force on atom j' in unit cell l' along the Cartesian direction β given a displacement in atom j in unit cell l along the Cartesian direction α. We use a series of single-point calculations using the LAMMPS code to obtain the forces on all atoms in the system given some set of displacement of the lattice from equilibrium. This set of displacements is generated by PHONOPY to include the 0.01 Å displacements of each atom in all three Cartesian directions. The phonon dispersion is shown explicitly in Fig. S7. Phonon eigenvectors are projected onto layers such that each band is colored by the square of this projection.

Phonon lifetimes from phonon-phonon perturbation theory calculation

We use the PHONO3PY (*55*, *65*) package to calculate the phonon lifetimes of phonon modes in $MoSe_2/WSe_2$ bilayer heterostructures at several high-symmetry stacking positions and interfacial twist angles. The phonon lifetimes are determined from the imaginary part of the phonon self-energy obtained from third-order force constants $\Phi_{\alpha\beta\gamma}\left(jl, j'l', j''l''\right)$ which are defined by the change in force on atom j'' in unit cell l'' along the Cartesian direction γ given a displacement in atom j in unit cell l along the Cartesian direction α and a displacement in atom j' in unit cell l' along the Cartesian direction β. These anharmonic, third-order terms are required to properly capture the three-phonon scattering process. To calculate the third-order force constant, we use PHONO3PY to generate an irreducible set of displacements of two atoms $(jl, j'l')$ with magnitude 0.03Å. We then perform many single-point calculations with LAMMPS to determine the change in force on the atom $(j''l'')$ due to these displacements. However, this step quickly becomes computationally expensive for large lattices as the size of the displacement set scales as $(3N_{\text{atoms}})^2$ and the size of the scattering matrix scales as $(3N_{\text{atoms}})^3$. To reduce the computational cost, we set a cutoff distance for the force. This sets the force between atoms beyond the cutoff to zero, reducing the number of force calculations to perform and the size of the scattering matrix in memory. We choose a cutoff distance of 7.5 Å, which accurately captures the intralayer interaction to second nearest-neighbor and the interlayer interaction to first nearest-neighbor. This approach accurately reproduces the phonon dispersion and phonon lifetimes of these bilayer TMD heterostructures. The generation of third-order force constants with force cutoff distances is performed by PHONO3PY.

Once constructed, we use the scattering matrix to compute the imaginary part of the self-energy which takes a form analogous to Fermi's golden rule,

$$\Gamma_{q,\nu}(\omega) = \frac{18\pi}{\hbar^2} \sum_{\mathbf{q}',\nu',\mathbf{q}'',\nu''} \left|\Phi_{-(\mathbf{q},\nu),(\mathbf{q},\nu,\mathbf{q},\nu)}\right|^2 \Big\{\left(n_{\mathbf{q}',\nu'} + n_{\mathbf{q}'',\nu''} + 1\right) \delta\left(\omega_{\mathbf{q},\nu} - \omega_{\mathbf{q}',\nu'} - \omega_{\mathbf{q}'',\nu''}\right) \tag{4}$$
$$+ \left(n_{\mathbf{q}',\nu'} - n_{\mathbf{q}'',\nu''}\right)\left[\delta\left(\omega_{\mathbf{q},\nu} + \omega_{\mathbf{q}',\nu'} - \omega_{\mathbf{q}'',\nu''}\right) - \left(\omega_{\mathbf{q},\nu} - \omega_{\mathbf{q}',\nu'} + \omega_{\mathbf{q}'',\nu''}\right)\right]\Big\},$$

where $\Gamma_{q,\nu}(\omega)$ corresponds to the phonon linewidth of the phonon mode $(\mathbf{q},\nu)$, ω is a phonon frequency, n is the Bose-Einstein occupation factor, and the phonon lifetime is calculated as

$$\tau_{\mathbf{q},\nu} = \frac{1}{2\Gamma_{\mathbf{q},\nu}\left(\omega_{\mathbf{q},\nu}\right)}. \tag{5}$$

The construction and solution of the imaginary part of the self-energy from the third-order force constants are handled by the PHOEBE package (*66*). We calculate phonon lifetimes on a 10x10x1 grid for a 4x4 supercell of $MoSe_2/WSe_2$ bilayer heterostructures in the 0˚, R_h^x stacking with 6 atoms in the primitive unit cell, and at finite interfacial twist angles 14˚ and 22˚ with 114 atoms and 42 atoms respectively. The lifetime for different phonons, resolved by their frequencies, is plotted in Fig. S8. Those phonons which are most likely to be produced in a non-thermal quantity during the initial hole relaxation after being generated by the pump are found in the shaded region.

Interfacial phonon-phonon scattering matrix elements in a layer-separable basis

The primary limitation of the above approach to quantify the non-thermal interfacial heat transfer is that the layer characteristics of the phonon modes involved in phonon-phonon scattering events are not well-defined. Naively, we might define the layer projection of a phonon in a bilayer system by the normalized displacement of the phonon modes within each layer: a phonon in layer, say, A would be a phonon whose normalized displacement is primarily in layer A. This would be an appropriate approach to calculate phonon-phonon interactions between phonon modes whose displacement is large, say, >99% in a single layer but fails when considering phonons that are nearly degenerate but very weakly hybridized due to the inherit degeneracy of the solutions. Any calculation of the interlayer heat transport involving an initial or final state that is layer-hybridized is therefore inaccurate and will underestimate the interlayer scattering time – and also will not be gauge independent for degenerate states.

To overcome this problem of characterization, we instead employ a layer-separable basis approach in which we rotate the phonon-phonon scattering matrix into a basis in which we can directly characterize all phonon modes by layer. We used a similar approach to Ouyang *et al.* (*56*), which calculated the phonon-phonon scattering elements by the off-diagonal components of the bilayer dynamical matrix rotated into a monolayer basis, but we extended this approach here to include the anharmonic effects from 3-phonon scattering processes. The separable monolayer basis, labeled below as AB, is constructed from the monolayer phonon modes, explicitly calculated on a single layer with the adjacent layer removed. These phonons are composed solely of displacements within a single layer and so are a good basis for isolating those phonon-phonon scattering events between layers. We then calculate the phonon-phonon scattering matrix elements as

$$\Phi^{AB}_{\lambda\lambda^{'}\lambda^{''}} = \sum_{\kappa\kappa^{'}\kappa^{''}} \sum_{\alpha\beta\gamma} F^{(3)}_{\alpha\beta\gamma}\left(\mathbf{q},\mathbf{q}^{'},\mathbf{q}^{''}\right) W^{AB}_{\alpha,\lambda} W^{AB}_{\beta,\lambda^{'}} W^{AB}_{\gamma,\lambda^{''}} \sqrt{\frac{\hbar}{2m_{\kappa}\omega^{AB}_{\lambda}}} \sqrt{\frac{\hbar}{2m_{\kappa^{'}}\omega^{AB}_{\lambda^{'}}}} \sqrt{\frac{\hbar}{2m_{\kappa^{''}}\omega^{AB}_{\lambda^{''}}}}, \tag{6}$$

where $F^{(3)}_{\alpha\beta\gamma}\left(q,q^{'},q''\right)$ is the third-order force constants in cartesian direction $\{\alpha,\beta,\gamma\} \in \{\hat{x},\hat{y},\hat{z}\}$ calculated on the full bilayer system using LAMMPS classical force fields, $W^{AB}_{\alpha,\lambda}$ are the eigenvectors of the interacting phonons in the monolayer basis with wave vector q and branch ν with $\lambda = (q,\nu)$ calculated on the separated monolayers using PHONOPY, and $\omega^{AB}_{\mathbf{q}j}$ are the monolayer phonon mode energies. In the separable monolayer basis, we can then easily extract those matrix elements which involves scattering from an initial phonon in layer A to two phonons, at least one of which is in the adjacent layer B. This selection procedure can be evaluated as

$$\Phi^{AB}_{\lambda_A \to \left\{\lambda^{'}_B \vee \lambda^{''}_B\right\}} = \Phi^{AB}_{\lambda\lambda^{'}\lambda^{''}} \delta_{\lambda,\lambda_A} \left[\delta_{\lambda^{'},\lambda^{''}_B} + \delta_{\lambda^{''},\lambda^{''}_B}\right]. \tag{7}$$

Using the identical Fermi's golden rule approach as previously employed, we can then calculate the interfacial phonon lifetimes using these phonon-phonon scattering matrix elements with the interlayer phonon scattering selected.

Examining non-equilibrium phonon characteristics

In the main text, we discussed a representative fast interfacial phonon scattering process which originates from an acoustic $\mathbf{q} \approx K$ phonon in the $MoSe_2$ layer. Our calculations show that the fast interlayer heat transfer time between WSe_2 and $MoSe_2$ originates from a $\mathbf{q} \approx K$ phonon with chiral displacement of the Se atoms about the xy-plane with a large out-of-plane displacement component throughout the oscillation period (Fig. S9). Previous time-domain reflectance experiments (TDTR) and DFT calculations show that the acoustic phonon modes with long-wavelength out-of-plane displacement carry a majority of heat between TMD layers (*67*). This particular phonon's large out-of-plane component could therefore similarly contribute to strong interlayer phonon coupling.

Lack of a substantial phonon highway in bilayers with different chalcogens

Our proposed mechanism depends on the presence of low-energy ZA phonons near the K point on both layers, and which are close in energy. Such a condition allows for the $\mathbf{q} \approx K$ phonon on the hot layer to be scattered to a $\mathbf{q} \approx K$ phonon on the cold layer with subsequent emission of a near-Γ, layer-hybridized acoustic phonon. The lowest-energy phonons at K primarily involve out-of-plane oscillation of the chalcogen atoms, and are hence nearly degenerate in bilayers with the same chalcogen atoms, such as bilayer $WSe_2/MoSe_2$, which we refer to as homochalcogen bilayer. Similarly, we argue that this mechanism should be substantially *reduced* on a bilayer where the two chalcogen atoms are different, which we refer to as a heterochalcogen bilayer.

To test the hypothesis that the fast interlayer heat transfer should be weak on a heterochalcogen bilayer, we compute the phonon band structure of bilayer MoS_2/WSe_2 in Fig. S10. As expected,

we find a much larger energy gap of 50 cm^{-1} between the two ZA acoustic bands at K at such a bilayer. We also compute the 3-phonon scattering processes in MoS_2/WSe_2 following the same approach as for $MoSe_2/WSe_2$ and find, as expected, longer scattering times associated with the interlayer phonon – about one order of magnitude larger in the heterochalcogen system compared to the homochalcogen bilayer (Fig. S11). We observe that there are still regions of fast interlayer scattering where the Mo acoustic bands overlap with the higher energy LA and optical bands of the W layer, consistent with our proposed mechanism.

MD simulation with nonthermal phonon distribution

For comparison to our perturbative theory approach, we perform an MD simulation on bilayer $MoSe_2/WSe_2$ with 0° twist angle to capture the temperature rise time of the WSe_2 layer, τ^W. However, instead of directly setting the temperature with a thermostat and thermalizing both layers, which generates a classical Maxwell-Boltzmann distribution of phonons, we add a *non-thermal initial distribution of phonons* to the $MoSe_2$ layer by freezing in a number of specific phonons. If we initialize a molecular dynamics simulation with such a non-thermal distribution of phonons, the system will dynamically evolve and natively include all orders of phonon-phonon interactions, though assuming classical phonon statistics.

We introduce these phonons by displacing atoms from their position at time $t = 0$ according to the phonon eigenvectors. We add all the thermal energy in the simulation to ZA phonons close to K to simulate a highly non-equilibrium phonon population. The specific mode was chosen from those displaying fast interlayer scattering, as observed in Fig S6. We evaluate the atomic displacement associated with a finite number of phonons as

$$\Delta\boldsymbol{\tau}_{p\kappa} = \left[\frac{\hbar}{2N_p M_\kappa \omega_{\mathbf{q}\nu}}(2n_{\mathbf{q}\nu,T} + 1)\right]^{\frac{1}{2}} \times 2\mathrm{Re}\left[e^{i\mathbf{q}\cdot\mathbf{R}_p}\mathbf{e}_{\kappa,\nu}(\mathbf{q})\right], \tag{8}$$

where $\Delta\boldsymbol{\tau}_{pk}$ represents the displacement of an atom κ having a mass of M_κ and situated in the unit cell with a lattice vector of $\mathbf{R}_p$. Here, N_p denotes the total number of unit cells within the supercell, $\mathbf{e}_{\kappa,\nu}(\mathbf{q})$ is the phonon polarization vector, normalized within the unit cell, with wave vector $\mathbf{q}$, band index ν, frequency $\omega_{\mathbf{q}\nu}$, and $n_{\mathbf{q}\nu,T}$ is the associated Bose-Einstein occupation at temperature *T*. In a supercell comprising 5400 atoms, we find that the effective number of acoustic phonons that need to be frozen in to increase the temperature of $MoSe_2$ by 50K results in an average displacement of 0.05 Å of the Se atoms. This value was determined by *post hoc* analysis of the MD simulation to obtain the desired temperature using a linear scale factor mimicking a larger phonon occupation factor.

For the first mode, we choose a phonon from the ZA band near the K point, which displays a short interlayer scattering time. By freezing this phonon in the Mo layer at $t = 0$ and allowing the system to evolve under constant volume and energy, we observe the W layer temperature rise time of $\tau_{\mathrm{MD}}^{\mathrm{W}} = 59 \pm 5$ ps (Fig. S10b). For completeness, we repeat this simulation for a phonon from the LA band at the K point, which shows a long interlayer scattering lifetime. In this case, we observe the W layer temperature rise time of $\tau_{\mathrm{MD}}^{\mathrm{W}} = 246 \pm 9$ ps (Fig. S10c). These results should be compared to the thermalization time of $\tau_{\mathrm{MD}} = 190 \pm 7$ ps reported in the main manuscript for a similar structure but for an initial thermal phonon within each layer (Fig. 3).

4. Supplementary Figures and Tables

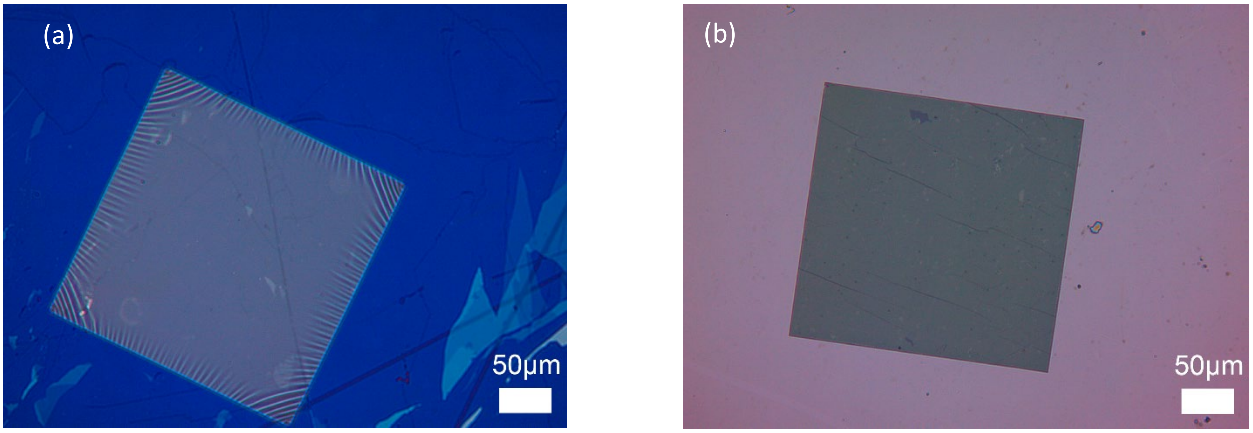

(a) **(b**

Fig. S1. Example Sample Images. (a) 4˚ MoS_2/WS_2 and (b) 7˚ $WSe_2/MoSe_2$ on TEM grid. The heterobilayer on the Si_3N_4 membrane window is the grey square in the center. The surrounding area is the Si grid. For each sample, the heterobilayer homogenously covers the entire window and exhibits minimal cracks or multilayer regions.

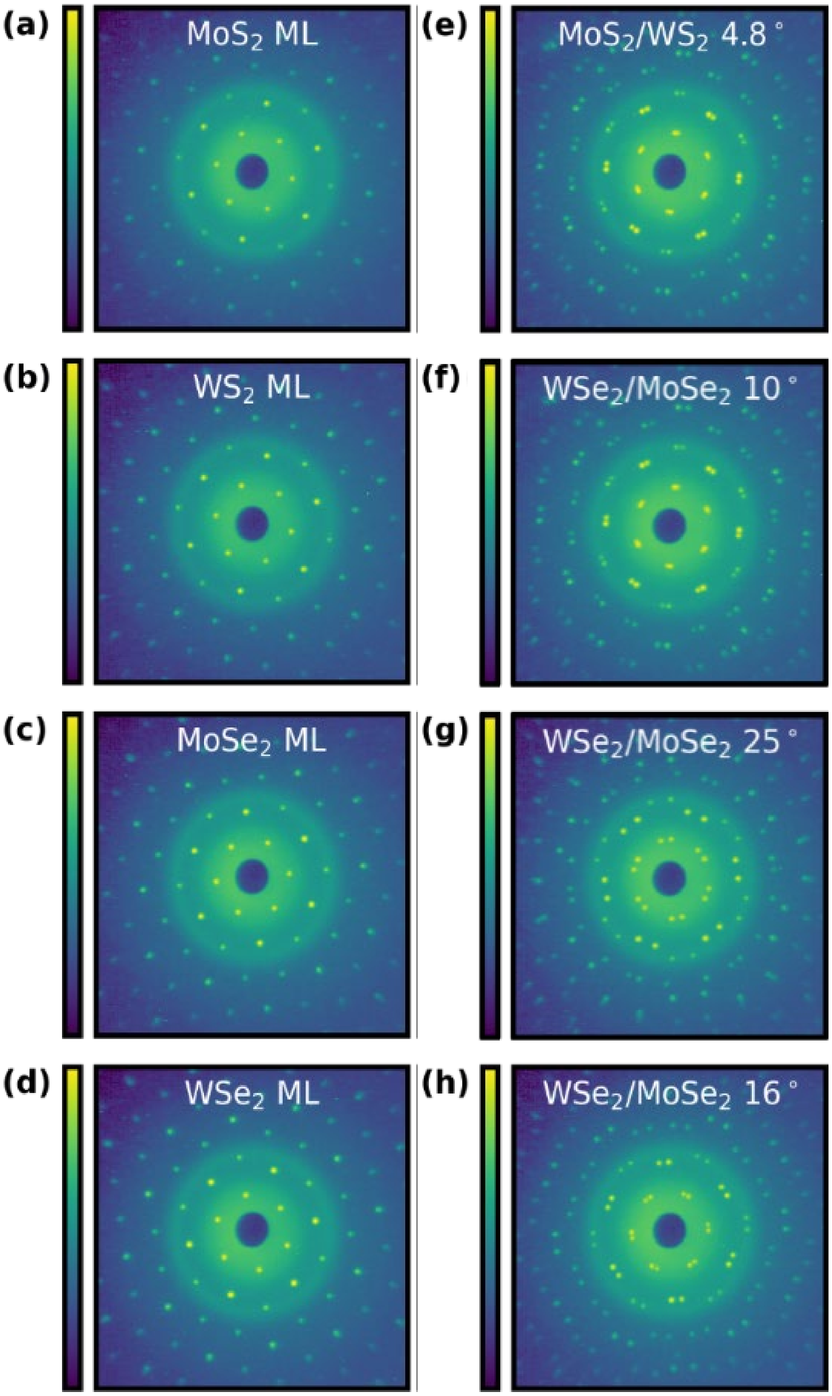

Fig. S2. Static Diffraction Images. (a-d) Isolated monolayers. (e-l) Twisted heterobilayers. Colorbars show relative intensity (arb. units).

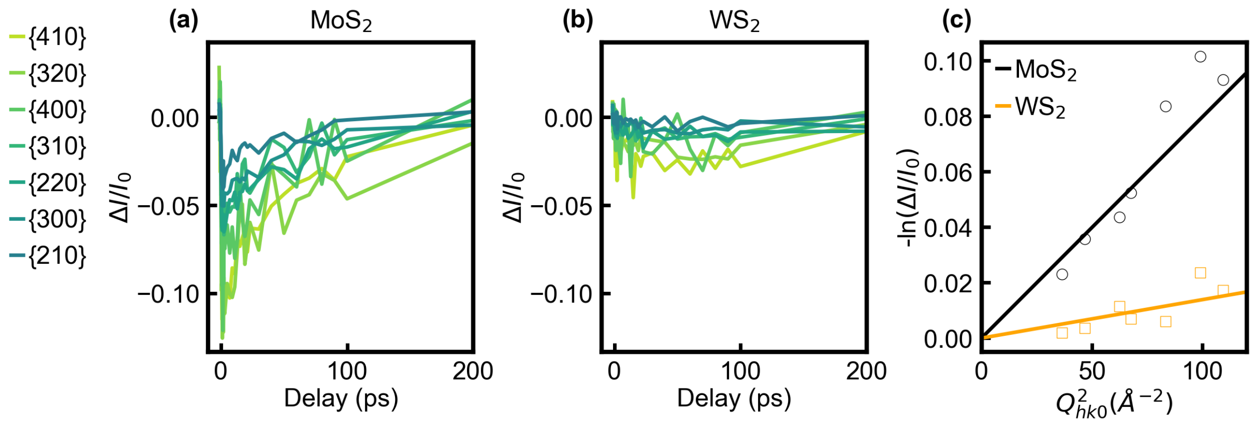

Fig. S3. Debye Waller Response for MoS_2/WS_2. (a)(b) Integrated Bragg peak intensities as a function of pump-probe delay time for Bragg peaks in the MoS_2 and WS_2 layers of a 4° MoS_2/WS_2 heterobilayer pumped on resonance with the MoS_2 A exciton with a fluence of 1 mJ/cm^2 at 50K. (c) Log intensity change for 7 orders of Bragg peaks at delay time of 12 ps, plotted against the reciprocal lattice vector of the Bragg peak squared, with the corresponding linear fit of the Debye-Waller model.

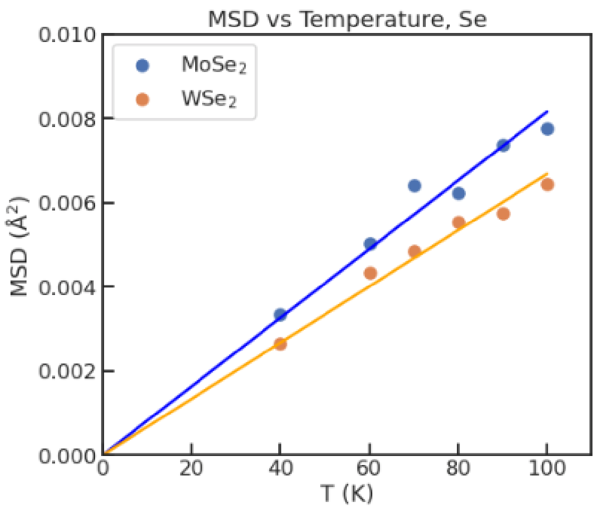

Fig. S4. MSD and Temperature. MSD of the $MoSe_2$ and WSe2 layers in a 7° $MoSe_2/WSe_2$ heterobilayer as a function of temperature. Fitted line shows expected linear relationship between MSD and temperature T.

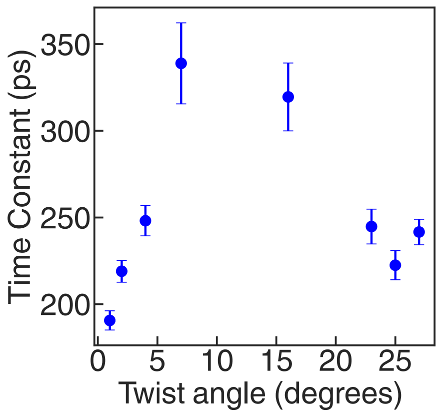

Fig. S5. Twist-angle dependent thermalization time of $MoSe_2/WSe_2$ from MD. Interlayer thermalization time of a $MoSe_2/WSe_2$ bilayer from MD simulations assuming each layer is individually in a local thermal equilibrium. The $MoSe_2$ and WSe_2 layers with different finite twist angles are thermalized to 100 K and 50 K respectively. At t = 0, the temperature constraints are released, and the system evolves under constant energy (NVE). The excess heat in the $MoSe_2$ layer transfers to the WSe_2, and the WSe_2 temperature rises until both layers reach equilibrium near 75 K. Thermalization time constants are extracted from single fit exponential to the temperature rise in the WSe_2.

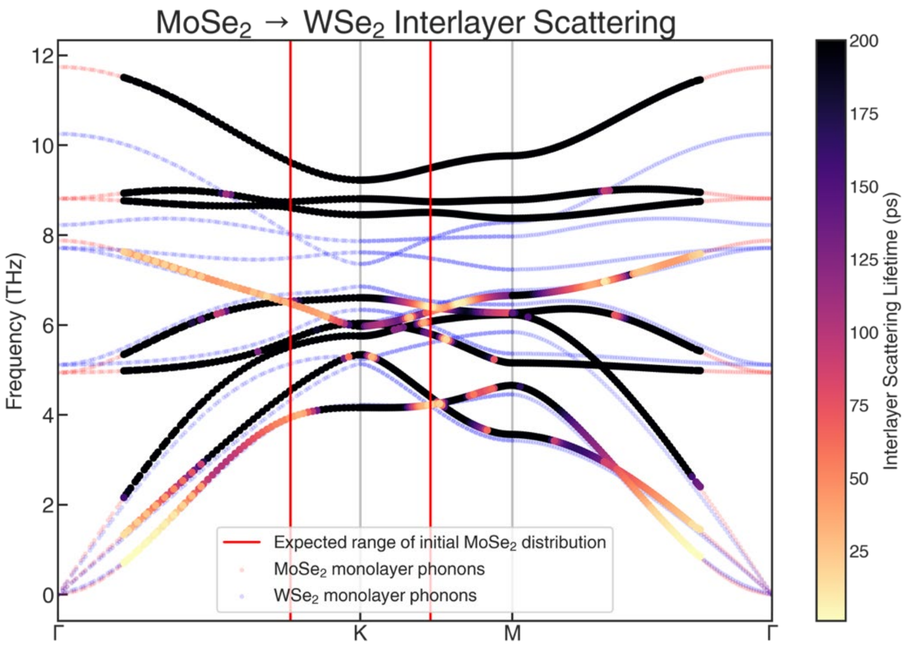

Fig. S6. Three-phonon scattering lifetimes for $MoSe_2/WSe_2$. Interlayer three-phonon scattering lifetime computed for an initial phonon of primarily $MoSe_2$ character. The phonon dispersion of the interacting system (thick lines) is also compared with that of an isolated layer of $MoSe_2$ (faint red dots) and WSe_2 (faint blue dots). Note that phonon dispersion associated with an isolated layer of $MoSe_2$ is nearly completely occluded by that of the interacting bilayer system. The fastest

interlayer scattering regions occur near the band crossings of $MoSe_2$ and WSe_2 phonons. The vertical red lines delimitate the expected range of initial phonon wavevectors originating from the hole relaxation in the $MoSe_2$ layer.

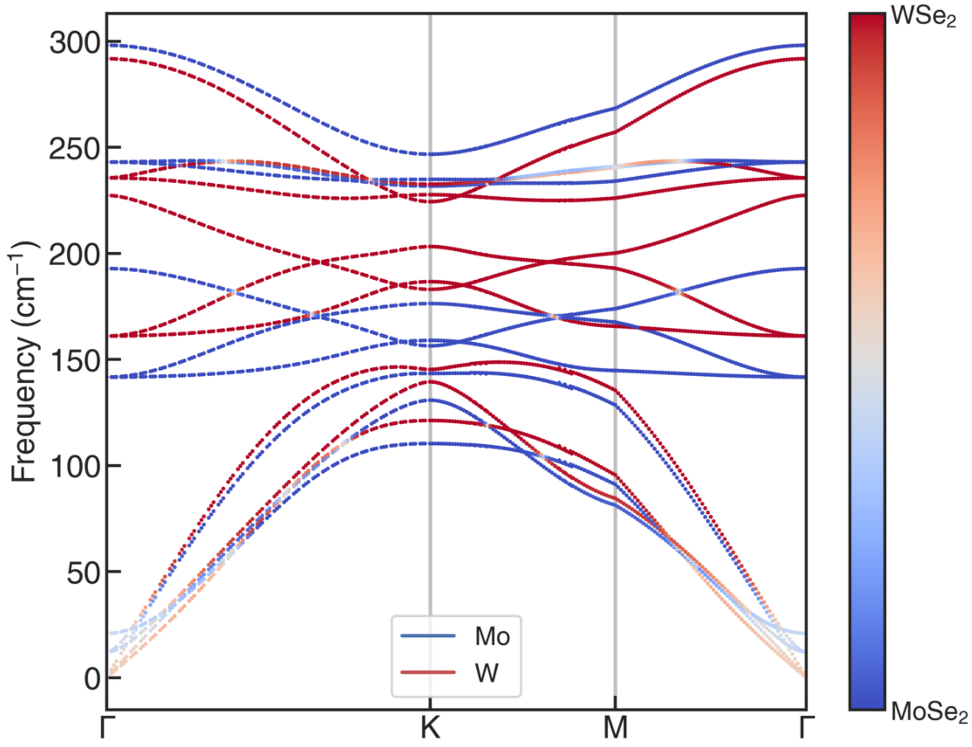

Fig. S7. $MoSe_2$/WSe_2 calculated phonon dispersion. Phonon dispersion for the $MoSe_2$/WSe_2 heterostructure projected onto the respective layers, computed with PHONOPY (*62*). Layer hybridization is minimum apart from band crossings and long wavelength acoustic modes. Note that the lowest-energy phonon bands near K are not strongly hybridized.

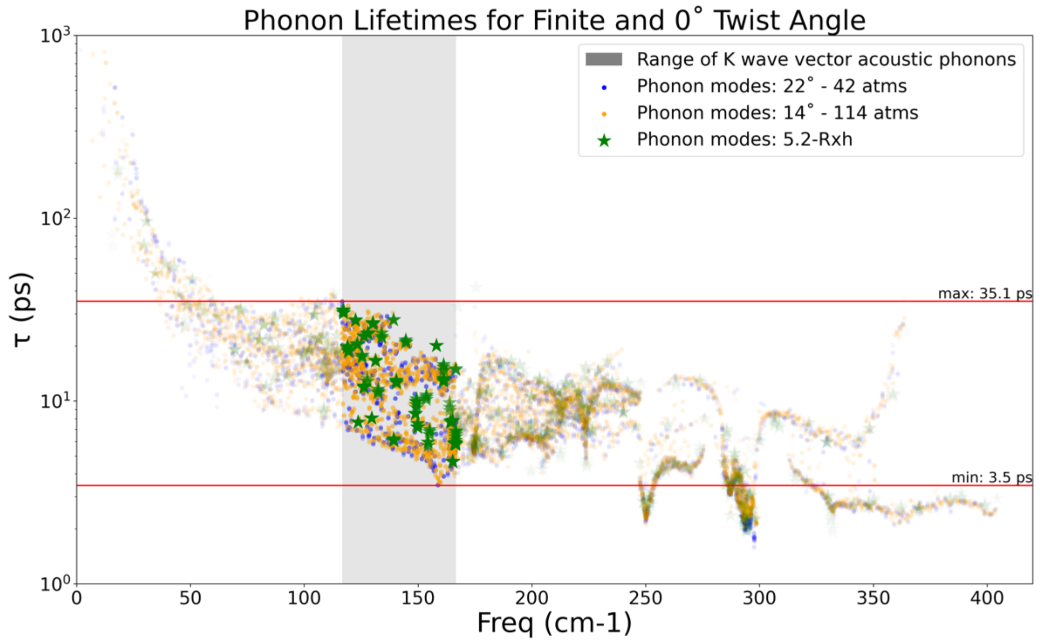

Fig. S8. Phonon lifetimes at different twist-angles. Phonon lifetimes at 300K for 0˚, 14˚, and 22˚ twisted $MoSe_2/WSe_2$ heterobilayers. Phonon lifetimes are calculated from the scattering matrix triplet elements between an initial phonon mode and two scattered phonon modes which preserve energy and crystal momentum. Grey region highlights those initial phonon modes which originate from acoustic modes near the K point of the BZ. Acoustic phonons between energies of 3.5 THz and 5 THz are primarily produced during the K-Γ hole scattering of the intra-to-interlayer $MoSe_2/WSe_2$. Minimum and maximum phonon lifetimes for this region is 3.5 ps and 35.1 ps, respectively.

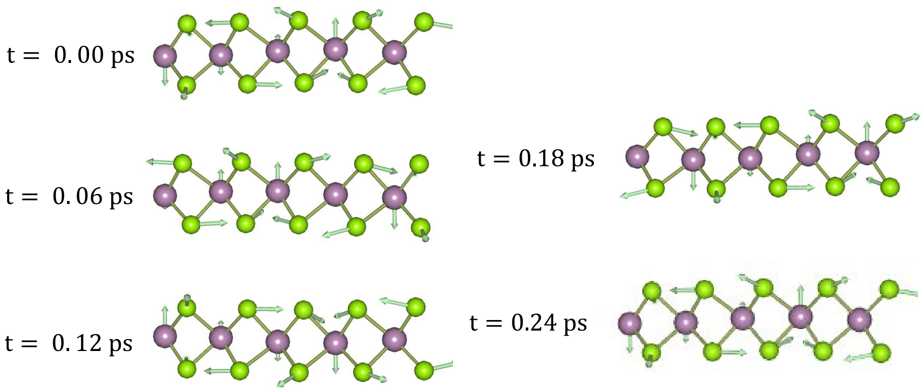

Fig. S9. Dynamics of a $MoSe_2$ phonon with fast interlayer phonon-phonon scattering. A particular $\mathbf{q} \approx K$ wavevector phonon of $MoSe_2$ along the K-M line which shows fast interlayer phonon-phonon scattering. The phonon has an energy of 17.4 meV (period of ~0.24 ps). The primary characteristic of this phonon is a chiral motion of the Se atoms along the in-plane direction and a displacement of the transition-metal atom along the out-of-plane direction. The phonon eigenvector was calculated using PHONOPY (*62*).

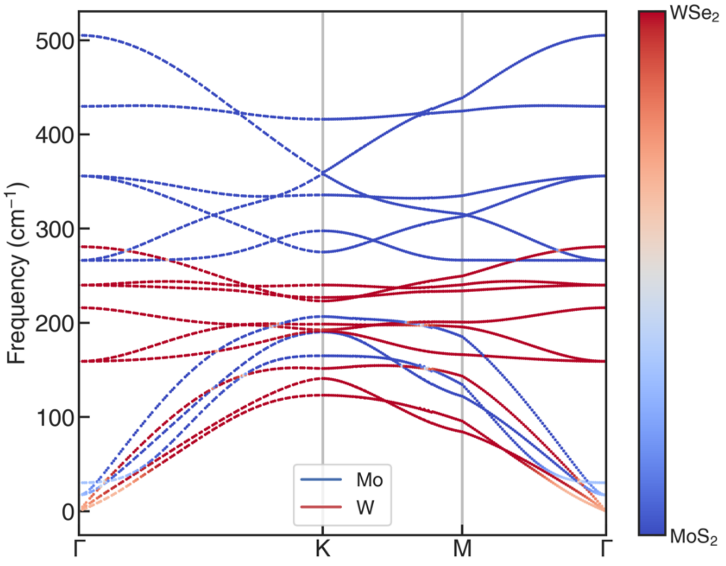

Fig. S10. MoS_2/WSe_2 calculated phonon dispersion. Phonon dispersions of bilayer MoS_2/WSe_2 computed with PHONOPY (*62*) using Stillinger-Weber and Kolmogorov-Crespi interatomic forces. Due to the lighter chalcogen, MoS_2 acoustic bands are approximately 50 cm^{-1} higher in energy than the WSe_2 acoustic bands and show little hybridization at K.

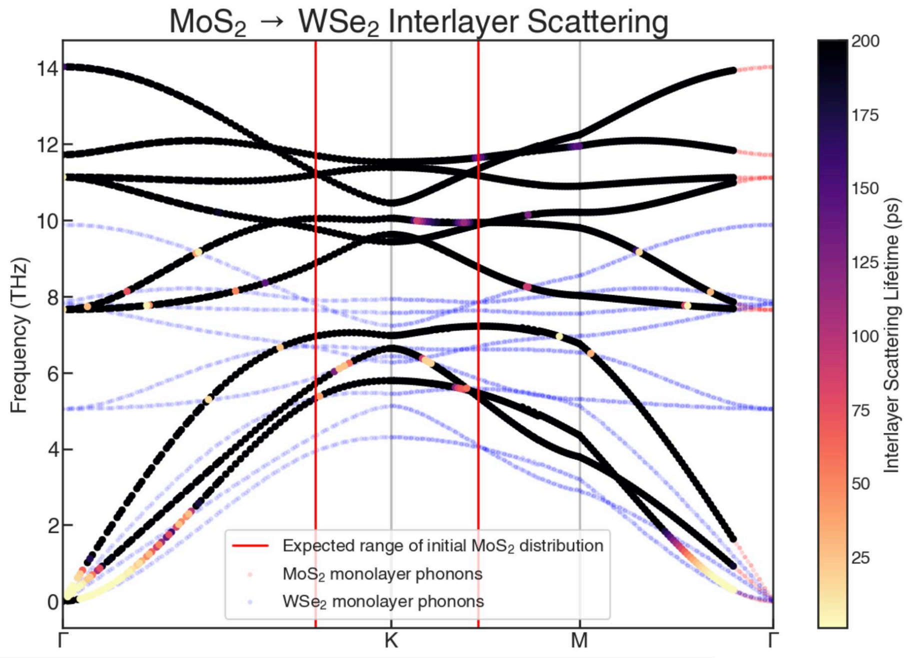

Fig. S11. Three-phonon scattering lifetimes for MoS_2/WSe_2. Interlayer three-phonon scattering lifetime computed for an initial phonon of primarily MoS_2 character in a MoS_2/WSe_2 heterochalcogen bilayer. The interlayer phonon lifetime of the initial Mo phonon (thick lines) is overlayed on the phonon dispersion of an isolated layer of $MoSe_2$ (faint red dots). The phonon dispersion of an isolated layer of WSe_2 (faint blue dots) is shown for comparison. The fastest interlayer scattering regions occur near the band crossings of MoS_2 and WSe_2 phonons, which are dramatically reduced along the ZA band compared to the $MoSe_2/WSe_2$ due to the presence of the energy gap between monolayer energies. The vertical red lines delimitate the expected range of initial phonon wavevectors originating from the hole relaxation in the MoS_2 layer.

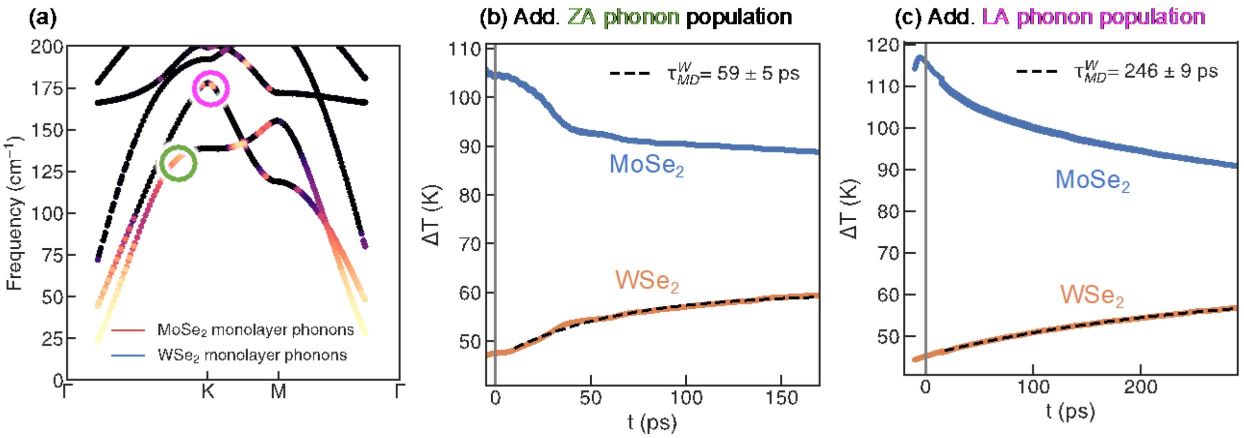

Fig. S12: **Interlayer thermalization time from MD simulations.** a) Interlayer thermalization time from MD simulations for the homochalcogen bilayer $MoSe_2/WSe_2$ with a 0˚ twist angle on a commensurate $R^X{}_H$ stacking. We chose two phonons to analyze: one from the ZA band with a short interlayer scattering time (green) and one from the LA band with a long interlayer scattering time (pink). b) Interfacial thermalization for a nonequilibrium phonon distribution. The $MoSe_2$ and WSe_2 layers are initially equilibrated with an NVT thermostat to 50 K and 50 K, respectively. At t = 0, a specific phonon mode from the ZA band close to K with a *short* interlayer scattering time is frozen into the Mo layer. The effective temperature of the layer is raised to 100K due to the non-equilibrium population. Immediately after, the temperature constraints are released, and the W layer temperature rises with a time scale $\tau_{MD} = 59 \pm 5$ ps, considerably faster than that found in the thermally distributed simulation. c) Similar to (b), but we initially freeze the highlighted phonon mode from the LA band with *long* interlayer scattering time. When the temperature constraints are released, the W layer temperature rises with a time scale $\tau_{MD} = 246 \pm 9$ ps.

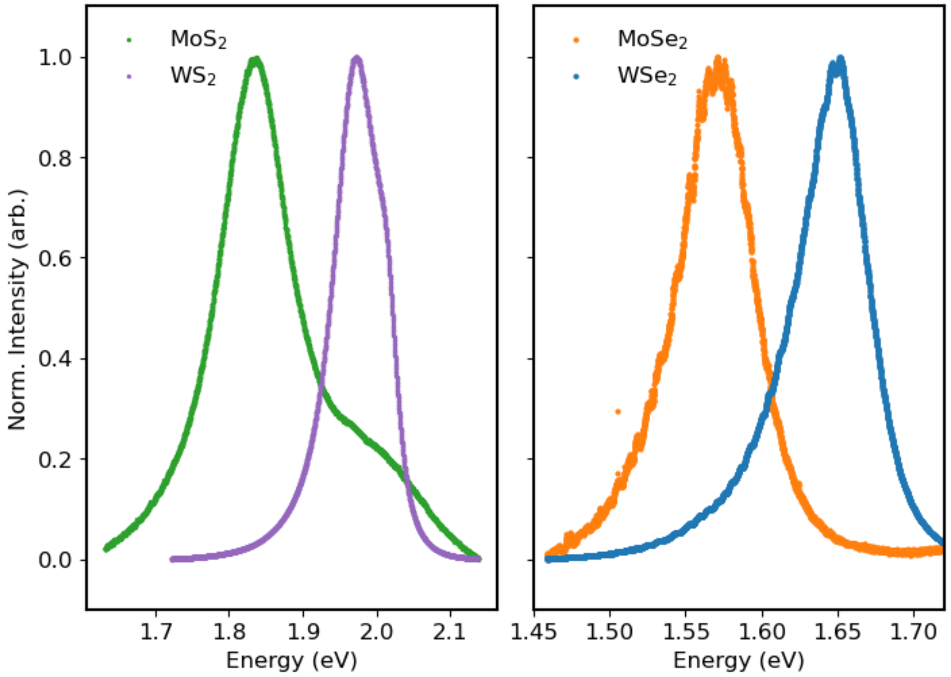

Fig. S13.Monolayer photoluminescence from measured samples. Photoluminescence spectra of monolayer regions in the 4° MoS_2/WS_2 heterobilayer and 25° $WSe_2/MoSe_2$ heterobilayer.

Table S1. Reciprocal lattice vectors used for Debye-Waller model. Different Bragg orders and the corresponding Q^2_{hk0} values for each monolayer. Lattice constants of 3.18 Å used for MoS_2 and WS_2 and 3.32 Å used for $MoSe_2$ and WSe_2 *(68)*.

Bragg Order {hk0}	MoS_2 (Å^{-2})	WS_2 (Å^{-2})	$MoSe_2$ (Å^{-2})	WSe_2 (Å^{-2})
{100}	5.21	5.21	4.78	4.78
{110}	15.62	15.62	14.33	14.33
{200}	20.82	20.82	19.10	19.10
{210}	36.44	36.44	33.43	33.43
{300}	46.85	46.85	42.98	42.98
{220}	62.46	62.46	57.31	57.31
{310}	67.67	67.67	62.08	62.08
{400}	83.28	83.28	76.41	76.41
{320}	98.90	98.90	90.74	90.74
{410}	109.31	109.31	100.29	100.29

REFERENCES AND NOTES

1. A. K. Geim, I. V. Grigorieva, Van der Waals heterostructures. *Nature* **499**, 419–425 (2013).

2. K. S. Novoselov, A. Mishchenko, A. Carvalho, A. H. Castro Neto, 2D materials and van der Waals heterostructures. *Science* **353**, aac9439 (2016).

3. E. M. Alexeev, D. A. Ruiz-Tijerina, M. Danovich, M. J. Hamer, D. J. Terry, P. K. Nayak, S. Ahn, S. Pak, J. Lee, J. I. Sohn, M. R. Molas, M. Koperski, K. Watanabe, T. Taniguchi, K. S. Novoselov, R. V. Gorbachev, H. S. Shin, V. I. Fal'ko, A. I. Tartakovskii, Resonantly hybridized excitons in moiré superlattices in van der Waals heterostructures. *Nature* **567**, 81–86 (2019).

4. P. Rivera, H. Yu, K. L. Seyler, N. P. Wilson, W. Yao, X. Xu, Interlayer valley excitons in heterobilayers of transition metal dichalcogenides. *Nat. Nanotechnol.* **13**, 1004–1015 (2018).

5. B. Huang, M. A. McGuire, A. F. May, D. Xiao, P. Jarillo-Herrero, X. Xu, Emergent phenomena and proximity effects in two-dimensional magnets and heterostructures. *Nat. Mater.* **19**, 1276–1289 (2020).

6. C.-H. Lee, G.-H. Lee, A. M. van der Zande, W. Chen, Y. Li, M. Han, X. Cui, G. Arefe, C. Nuckolls, T. F. Heinz, J. Guo, J. Hone, P. Kim, Atomically thin p–N junctions with van der Waals heterointerfaces. *Nat. Nanotechnol.* **9**, 676–681 (2014).

7. Y. Liu, N. O. Weiss, X. Duan, H. C. Cheng, Y. Huang, X. Duan, Van der Waals heterostructures and devices. *Nat. Rev. Mater.* **1**, 16042 (2016).

8. C. Jin, E. Y. Ma, O. Karni, E. C. Regan, F. Wang, T. F. Heinz, Ultrafast dynamics in van der Waals heterostructures. *Nat. Nanotechnol.* **13**, 994–1003 (2018).

9. Y. Liu, X. Duan, H.-J. Shin, S. Park, Y. Huang, X. Duan, Promises and prospects of two-dimensional transistors. *Nature* **591**, 43–53 (2021).

10. S.-J. Liang, B. Cheng, X. Cui, F. Miao, Van der Waals heterostructures for high-performance device applications: Challenges and opportunities. *Adv. Mater.* **32**, e1903800 (2020).

11. A. Ciarrocchi, F. Tagarelli, A. Avsar, A. Kis, Excitonic devices with van der Waals heterostructures: Valleytronics meets twistronics. *Nat. Rev. Mater.* **7**, 449–464 (2022).

12. Q. Zeng, Z. Liu, Novel optoelectronic devices: Transition-metal-dichalcogenide-based 2D heterostructures. *Adv. Electron. Mater.* **4**, 1700335 (2018).

13. K. Tran, G. Moody, F. Wu, X. Lu, J. Choi, K. Kim, A. Rai, D. A. Sanchez, J. Quan, A. Singh, J. Embley, A. Zepeda, M. Campbell, T. Autry, T. Taniguchi, K. Watanabe, N. Lu, S. K. Banerjee, K. L. Silverman, S. Kim, E. Tutuc, L. Yang, A. H. MacDonald, X. Li, Evidence for moiré excitons in van der Waals heterostructures. *Nature* **567**, 71–75 (2019).

14. K. L. Seyler, P. Rivera, H. Yu, N. P. Wilson, E. L. Ray, D. G. Mandrus, J. Yan, W. Yao, X. Xu, Signatures of moiré-trapped valley excitons in $MoSe_2$/WSe_2 heterobilayers. *Nature* **567**, 66–70 (2019).

15. P. Rivera, J. R. Schaibley, A. M. Jones, J. S. Ross, S. Wu, G. Aivazian, P. Klement, K. Seyler, G. Clark, N. J. Ghimire, J. Yan, D. G. Mandrus, W. Yao, X. Xu, Observation of long-lived interlayer excitons in monolayer $MoSe_2$–WSe_2 heterostructures. *Nat. Commun.* **6**, 6242 (2015).

16. C. Jin, E. C. Regan, A. Yan, M. I. B. Utama, D. Wang, S. Zhao, Y. Qin, S. Yang, Z. Zheng, S. Shi, K. Watanabe, T. Taniguchi, S. Tongay, A. Zettl, F. Wang, Observation of moiré excitons in WSe_2/WS_2 heterostructure superlattices. *Nature* **567**, 76–80 (2019).

17. K. Tran, J. Choi, A. Singh, Moiré and beyond in transition metal dichalcogenide twisted bilayers. *2D Mater.* **8**, 022002 (2020).

18. D. Huang, J. Choi, C.-K. Shih, X. Li, Excitons in semiconductor moiré superlattices. *Nat. Nanotechnol.* **17**, 227–238 (2022).

19. K. F. Mak, J. Shan, Photonics and optoelectronics of 2D semiconductor transition metal dichalcogenides. *Nat. Photonics.* **10**, 216–226 (2016).

20. E. C. Regan, D. Wang, E. Y. Paik, Y. Zeng, L. Zhang, J. Zhu, A. H. MacDonald, H. Deng, F. Wang, Emerging exciton physics in transition metal dichalcogenide heterobilayers. *Nat. Rev. Mater.* **7**, 778–795 (2022).

21. L. Yuan, B. Zheng, J. Kunstmann, T. Brumme, A. B. Kuc, C. Ma, S. Deng, D. Blach, A. Pan, L. Huang, Twist-angle-dependent interlayer exciton diffusion in WS_2–WSe_2 heterobilayers. *Nat. Mater.* **19**, 617–623 (2020).

22. Y. Zhu, W.-H. Fang, A. Rubio, R. Long, O. V. Prezhdo, The twist angle has weak influence on charge separation and strong influence on recombination in the MoS_2/WS_2 bilayer: Ab initio quantum dynamics. *J. Mater. Chem. A* **10**, 8324–8333 (2022).

23. R. Long, O. V. Prezhdo, quantum coherence facilitates efficient charge separation at a MoS_2/$MoSe_2$ van der Waals junction. *Nano Lett.* **16**, 1996–2003 (2016).

24. Y. Wang, Z. Wang, W. Yao, G.-B. Liu, H. Yu, Interlayer coupling in commensurate and incommensurate bilayer structures of transition-metal dichalcogenides. *Phys. Rev. B.* **95**, 115429 (2017).

25. G. Meneghini, S. Brem, E. Malic, Ultrafast phonon-driven charge transfer in van der Waals heterostructures. *Nat. Sci.* **2**, e20220014 (2022).

26. Q. Zheng, W. A. Saidi, Y. Xie, Z. Lan, O. V. Prezhdo, H. Petek, J. Zhao, Phonon-assisted ultrafast charge transfer at van der Waals heterostructure interface. *Nano Lett.* **17**, 6435–6442 (2017).

27. Q. Zheng, Y. Xie, Z. Lan, O. V. Prezhdo, W. A. Saidi, J. Zhao, Phonon-coupled ultrafast interlayer charge oscillation at van der Waals heterostructure interfaces. *Phys. Rev. B.* **97**, 205417 (2018).

28. Z. Wang, P. Altmann, C. Gadermaier, Y. Yang, W. Li, L. Ghirardini, C. Trovatello, M. Finazzi, L. Duò, M. Celebrano, R. Long, D. Akinwande, O. V. Prezhdo, G. Cerullo, S. Dal Conte, Phonon-mediated interlayer charge separation and recombination in a $MoSe_2$/WSe_2 heterostructure. *Nano Lett.* **21**, 2165–2173 (2021).

29. X.-K. Chen, Y.-J. Zeng, K.-Q. Chen, Thermal transport in two-dimensional heterostructures. *Front. Mater.* **7**, 578791 (2020).

30. Z.-Y. Ong, M.-H. Bae, Energy dissipation in van der Waals 2D devices. *2D Mater.* **6**, 032005 (2019).

31. S. E. Kim, F. Mujid, A. Rai, F. Eriksson, J. Suh, P. Poddar, A. Ray, C. Park, E. Fransson, Y. Zhong, D. A. Muller, P. Erhart, D. G. Cahill, J. Park, Extremely anisotropic van der Waals thermal conductors. *Nature* **597**, 660–665 (2021).

32. B. Amin, N. Singh, U. Schwingenschlögl, Heterostructures of transition metal dichalcogenides. *Phys. Rev. B.* **92**, 075439 (2015).

33. V. O. Özçelik, J. G. Azadani, C. Yang, S. J. Koester, T. Low, Band alignment of two-dimensional semiconductors for designing heterostructures with momentum space matching. *Phys. Rev. B.* **94**, 035125 (2016).

34. X. Hong, J. Kim, S.-F. Shi, Y. Zhang, C. Jin, Y. Sun, S. Tongay, J. Wu, Y. Zhang, F. Wang, Ultrafast charge transfer in atomically thin MoS_2/WS_2 heterostructures. *Nat. Nanotechnol.* **9**, 682–686 (2014).

35. Z. Ji, H. Hong, J. Zhang, Q. Zhang, W. Huang, T. Cao, R. Qiao, C. Liu, J. Liang, C. Jin, L. Jiao, K. Shi, S. Meng, K. Liu, Robust stacking-independent ultrafast charge transfer in MoS_2/WS_2 bilayers. *ACS Nano* **11**, 12020–12026 (2017).

36. V. R. Policht, M. Russo, F. Liu, C. Trovatello, M. Maiuri, Y. Bai, X. Zhu, S. Dal Conte, G. Cerullo, Dissecting interlayer hole and electron transfer in transition metal dichalcogenide heterostructures via two-dimensional electronic spectroscopy. *Nano Lett.* **21**, 4738–4743 (2021).

37. H. Zhu, J. Wang, Z. Gong, Y. D. Kim, J. Hone, X.-Y. Zhu, Interfacial charge transfer circumventing momentum mismatch at two-dimensional van der Waals heterojunctions. *Nano Lett.* **17**, 3591–3598 (2017).

38. E. M. Mannebach, R. Li, K.-A. Duerloo, C. Nyby, P. Zalden, T. Vecchione, F. Ernst, A. H. Reid, T. Chase, X. Shen, S. Weathersby, C. Hast, R. Hettel, R. Coffee, N. Hartmann, A. R. Fry, Y. Yu, L. Cao, T. F. Heinz, E. J. Reed, H. A. Dürr, X. Wang, A. M. Lindenberg, Dynamic structural response and deformations of monolayer MoS_2 visualized by femtosecond electron diffraction. *Nano Lett.* **15**, 6889–6895 (2015).

39. T. L. Britt, Q. Li, L. P. René de Cotret, N. Olsen, M. Otto, S. A. Hassan, M. Zacharias, F. Caruso, X. Zhu, B. J. Siwick, Direct view of phonon dynamics in atomically thin MoS_2. *Nano Lett.* **22**, 4718–4724 (2022).

40. M.-F. Lin, V. Kochat, A. Krishnamoorthy, L. Bassman, C. Weninger, Q. Zheng, X. Zhang, A. Apte, C. S. Tiwary, X. Shen, R. Li, R. Kalia, P. Ajayan, A. Nakano, P. Vashishta, F. Shimojo, X. Wang, D. M. Fritz, U. Bergmann, Ultrafast non-radiative dynamics of atomically thin $MoSe_2$. *Nat. Commun.* **8**, 1745 (2017).

41. D. Luo, J. Tang, X. Shen, F. Ji, J. Yang, S. Weathersby, M. E. Kozina, Z. Chen, J. Xiao, Y. Ye, T. Cao, G. Zhang, X. Wang, A. M. Lindenberg, Twist-angle-dependent ultrafast charge transfer in MoS_2-graphene van der Waals heterostructures. *Nano Lett.* **21**, 8051–8057 (2021).

42. D. Luo, D. Hui, B. Wen, R. Li, J. Yang, X. Shen, A. H. Reid, S. Weathersby, M. E. Kozina, S. Park, Y. Ren, T. D. Loeffler, S. K. R. S. Sankaranarayanan, M. K. Y. Chan, X. Wang, J. Tian, I. Arslan, X. Wang, T. Rajh, J. Wen, Ultrafast formation of a transient two-dimensional diamondlike structure in twisted bilayer graphene. *Phys. Rev. B.* **102**, 155431 (2020).

43. A. Sood, J. B. Haber, J. Carlström, E. A. Peterson, E. Barre, J. D. Georgaras, A. H. M. Reid, X. Shen, M. E. Zajac, E. C. Regan, J. Yang, T. Taniguchi, K. Watanabe, F. Wang, X. Wang, J. B. Neaton, T. F. Heinz, A. M. Lindenberg, F. H. da Jornada, A. Raja, Bidirectional phonon emission in two-dimensional heterostructures triggered by ultrafast charge transfer. *Nat. Nanotechnol.* **18**, 29–35 (2023).

44. S. P. Weathersby, G. Brown, M. Centurion, T. F. Chase, R. Coffee, J. Corbett, J. P. Eichner, J. C. Frisch, A. R. Fry, M. Gühr, N. Hartmann, C. Hast, R. Hettel, R. K. Jobe, E. N. Jongewaard, J. R. Lewandowski, R. K. Li, A. M. Lindenberg, I. Makasyuk, J. E. May, D. McCormick, M. N. Nguyen, A. H. Reid, X. Shen, K. Sokolowski-Tinten, T. Vecchione, S. L. Vetter, J. Wu, J. Yang, H. A. Dürr, X. J. Wang, Mega-electron-volt ultrafast electron diffraction at SLAC National Accelerator Laboratory. *Rev. Sci. Instrum.* **86**, 073702 (2015).

45. X. Shen, R. K. Li, U. Lundström, T. J. Lane, A. H. Reid, S. P. Weathersby, X. J. Wang, Femtosecond mega-electron-volt electron microdiffraction. *Ultramicroscopy* **184**, 172–176 (2018).

46. F. Liu, W. Wu, Y. Bai, S. H. Chae, Q. Li, J. Wang, J. Hone, X.-Y. Zhu, Disassembling 2D van der Waals crystals into macroscopic monolayers and reassembling into artificial lattices. *Science* **367**, 903–906 (2020).

47. S. H. Simon, *The Oxford Solid State Basics* (Oxford Univ. Press, 2013).

48. C. J. R. Duncan, M. Kaemingk, W. H. Li, M. B. Andorf, A. C. Bartnik, A. Galdi, M. Gordon, C. A. Pennington, I. V. Bazarov, H. J. Zeng, F. Liu, D. Luo, A. Sood, A. M. Lindenberg, M. W. Tate, D. A. Muller, J. Thom-Levy, S. M. Gruner, J. M. Maxson, Multi-scale time-resolved electron diffraction: A case study in moiré materials. *Ultramicroscopy* **253**, 113771 (2023).

49. I.-C. Tung, A. Krishnamoorthy, S. Sadasivam, H. Zhou, Q. Zhang, K. L. Seyler, G. Clark, E. M. Mannebach, C. Nyby, F. Ernst, D. Zhu, J. M. Glownia, M. E. Kozina, S. Song, S. Nelson, H. Kumazoe, F. Shimojo, R. K. Kalia, P. Vashishta, P. Darancet, T. F. Heinz, A. Nakano, X. Xu, A. M. Lindenberg, H. Wen, Anisotropic structural dynamics of monolayer crystals revealed by femtosecond surface X-ray scattering. *Nat. Photonics.* **13**, 425–430 (2019).

50. N. R. Wilson, P. V. Nguyen, K. Seyler, P. Rivera, A. J. Marsden, Z. P. L. Laker, G. C. Constantinescu, V. Kandyba, A. Barinov, N. D. M. Hine, X. Xu, D. H. Cobden, Determination of band offsets, hybridization, and exciton binding in 2D semiconductor heterostructures. *Sci. Adv.* **3**, e1601832 (2017).

51. F. Caruso, Nonequilibrium lattice dynamics in monolayer MoS_2. *J. Phys. Chem. Lett.* **12**, 1734–1740 (2021).

52. A. P. Thompson, H. M. Aktulga, R. Berger, D. S. Bolintineanu, W. M. Brown, P. S. Crozier, P. J. In‘t Veld, A. Kohlmeyer, S. G. Moore, T. D. Nguyen, R. Shan, M. J. Stevens, J. Tranchida, C. Trott, S. J. Plimpton, LAMMPS - a flexible simulation tool for particle-based materials modeling at the atomic, meso, and continuum scales. *Comput. Phys. Commun.* **271**, 108171 (2022).

53. A. Krishnamoorthy, P. Rajak, P. Norouzzadeh, D. J. Singh, R. K. Kalia, A. Nakano, P. Vashishta, Thermal conductivity of MoS_2 monolayers from molecular dynamics simulations. *AIP Adv.* **9**, 035042 (2019).

54. L. Waldecker, R. Bertoni, H. Hübener, T. Brumme, T. Vasileiadis, D. Zahn, A. Rubio, R. Ernstorfer, Momentum-resolved view of electron-phonon coupling in Multilayer WSe_2. *Phys. Rev. Lett.* **119**, 036803 (2017).

55. A. Togo, L. Chaput, I. Tanaka, Distributions of phonon lifetimes in Brillouin zones. *Phys. Rev. B.* **91**, 094306 (2015).

56. W. Ouyang, H. Qin, M. Urbakh, O. Hod, Controllable thermal conductivity in twisted homogeneous interfaces of graphene and hexagonal boron nitride. *Nano Lett.* **20**, 7513–7518 (2020).

57. A. B. Kuzmenko, Kramers–Kronig constrained variational analysis of optical spectra. *Rev. Sci. Instrum.* **76**, 083108 (2005).

58. P. Giannozzi, O. Andreussi, T. Brumme, O. Bunau, M. Buongiorno Nardelli, M. Calandra, R. Car, C. Cavazzoni, D. Ceresoli, M. Cococcioni, N. Colonna, I. Carnimeo, A. Dal Corso, S. de Gironcoli, P. Delugas, R. A. DiStasio, A. Ferretti, A. Floris, G. Fratesi, G. Fugallo, R. Gebauer, U. Gerstmann, F. Giustino, T. Gorni, J. Jia, M. Kawamura, H.-Y. Ko, A. Kokalj, E. Küçükbenli, M. Lazzeri, M. Marsili, N. Marzari, F. Mauri, N. L. Nguyen, H.-V. Nguyen, A. Otero-de-la-Roza, L. Paulatto, S. Poncé, D. Rocca, R. Sabatini, B. Santra, M. Schlipf, A. P. Seitsonen, A. Smogunov, I. Timrov, T. Thonhauser, P. Umari, N. Vast, X. Wu, S. Baroni, Advanced capabilities for materials modelling with Quantum ESPRESSO. *J. Phys. Condens. Matter* **29**, 465901 (2017).

59. J. P. Perdew, K. Burke, M. Ernzerhof, Generalized gradient approximation made simple. *Phys. Rev. Lett.* **77**, 3865–3868 (1996).

60. M. Dion, H. Rydberg, E. Schröder, D. C. Langreth, B. I. Lundqvist, Van der Waals density functional for general geometries. *Phys. Rev. Lett.* **92**, 246401 (2004).

61. V. R. Cooper, Van der Waals density functional: An appropriate exchange functional. *Phys. Rev. B* **81**, 161104 (2010).

62. A. Togo, I. Tanaka, First principles phonon calculations in materials science. *Scr. Mater.* **108**, 1–5 (2015).

63. J.-W. Jiang, Parametrization of Stillinger–Weber potential based on valence force field model: Application to single-layer MoS_2 and black phosphorus. *Nanotechnology* **26**, 315706 (2015).

64. M. H. Naik, I. Maity, P. K. Maiti, M. Jain, Kolmogorov–Crespi potential for multilayer transition-metal dichalcogenides: Capturing structural transformations in moiré superlattices. *J. Phys. Chem. C* **123**, 9770–9778 (2019).

65. A. Togo, First-principles Phonon Calculations with Phonopy and Phono3py. *J. Physical Soc. Japan* **92**, 012001 (2023).

66. A. Cepellotti, J. Coulter, A. Johansson, N. S. Fedorova, B. Kozinsky, Phoebe: A high-performance framework for solving phonon and electron Boltzmann transport equations. *J. Phys. Mater.* **5**, 035003 (2022).

67. A. Sood, F. Xiong, S. Chen, R. Cheaito, F. Lian, M. Asheghi, Y. Cui, D. Donadio, K. E. Goodson, E. Pop, Quasi-ballistic thermal transport across MoS_2 thin films. *Nano Lett.* **19**, 2434–2442 (2019).

68. H. L. Zhuang, R. G. Hennig, Computational search for single-layer transition-metal dichalcogenide photocatalysts. *J. Phys. Chem. C* **117**, 20440–20445 (2013).
